# Combining Stable Isotope Labeling and Candidate Substrate–Product Pair Networks Reveals Lignan, Oligolignol, and Chicoric Acid Biosynthesis in Flax Seedlings (*Linum usitatissimum* L.)

**DOI:** 10.3390/plants14152371

**Published:** 2025-08-01

**Authors:** Benjamin Thiombiano, Ahlam Mentag, Manon Paniez, Romain Roulard, Paulo Marcelo, François Mesnard, Rebecca Dauwe

**Affiliations:** 1 Unité de Recherche BIOPI, UMR Transfrontalière BioEcoAgro, Université de Picardie Jules Verne, 80000 Amiens, France; benjamin.thiombiano@syngenta.com (B.T.); ahlam.mentag@u-picardie.fr (A.M.); manon.paniez@etud.u-picardie.fr (M.P.); romain.roulard@u-picardie.fr (R.R.); francois.mesnard@u-picardie.fr (F.M.); 2 Plateforme Ingénierie Cellulaire et Analyses des Protéines, Université de Picardie Jules Verne, 80000 Amiens, France; paulo.marcelo@u-picardie.fr

**Keywords:** flax (*Linum usitatissimum* L.), stable isotope labeling (SIL), phenylpropanoid metabolism, candidate substrate–product pair (CSPP) networks, mass spectrometry, tartaric acid esters

## Abstract

Functional foods like flax (*Linum usitatissimum* L.) are rich sources of specialized metabolites that contribute to their nutritional and health-promoting properties. Understanding the biosynthesis of these compounds is essential for improving their quality and potential applications. However, dissecting complex metabolic networks in plants remains challenging due to the dynamic nature and interconnectedness of biosynthetic pathways. In this study, we present a synergistic approach combining stable isotopic labeling (SIL), Candidate Substrate–Product Pair (CSPP) networks, and a time-course study with high temporal resolution to reveal the biosynthetic fluxes shaping phenylpropanoid metabolism in young flax seedlings. By feeding the seedlings with ^13^C_3_-*p*-coumaric acid and isolating isotopically labeled metabolization products prior to the construction of CSPP networks, the biochemical validity of the connections in the network was supported by SIL, independent of spectral similarity or abundance correlation. This method, in combination with multistage mass spectrometry (MS^n^), allowed confident structural proposals of lignans, neolignans, and hydroxycinnamic acid conjugates, including the presence of newly identified chicoric acid and related tartaric acid esters in flax. High-resolution time-course analyses revealed successive waves of metabolite formation, providing insights into distinct biosynthetic fluxes toward lignans and early lignification intermediates. No evidence was found here for the involvement of chlorogenic or caftaric acid intermediates in chicoric acid biosynthesis in flax, as has been described in other species. Instead, our findings suggest that in flax seedlings, chicoric acid is synthesized through successive hydroxylation steps of *p*-coumaroyl tartaric acid esters. This work demonstrates the power of combining SIL and CSPP strategies to uncover novel metabolic routes and highlights the nutritional potential of flax sprouts rich in chicoric acid.

## 1. Introduction

Flax (*Linum usitatissimum* L.) has been cultivated for millennia for its fibers and seed oil. More recently, interest has grown in its value as a source of bioactive compounds for pharmaceuticals and functional foods, including polyunsaturated fatty acids, digestible proteins, mucilage, and phenolics [1]. Plant phenolics form a structurally diverse group of specialized metabolites derived from multiple branches of phenylpropanoid metabolism. In most higher plants, the major phenylpropanoid flux leads to lignin, which reinforces cell walls, supports water transport, and provides a barrier against pathogens [2]. Beyond lignin, phenylpropanoids play essential roles in UV protection and responses to abiotic and biotic stress [3] and contribute to human health as antioxidants and anti-inflammatory agents in functional foods.

In flax, secoisolariciresinol diglucoside (SDG) is the most extensively characterized phenolic, accumulating in seed hulls and making flaxseeds one of the richest natural sources of lignans [4,5,6]. However, phenolic metabolism extends beyond the seed. Studies have identified phenolic accumulation in leaves, flowers, stems, fibers, straw, and seedlings [7,8,9,10,11]. Flax seedlings, also referred to as flax sprouts, have emerged as promising functional foods, showing increased levels of vitamin C, total phenolics, flavonoids, and antioxidant activity during early germination [8,12,13].

Until now, the analyses of phenolics in flax sprouts have largely targeted SDG and a few phenolic acids, typically relying on external standards for compound identification and quantification [8,12]. Yet, phenolics are often subject to complex glycosylation and acylation, leading to structurally diverse, multiply conjugated metabolites that are typically not represented in standard compound databases [14]. Therefore, untargeted profiling of flax sprout phenolics requires approaches capable of de novo structure elucidation.

In LC–MS-based metabolomics, low-energy collision-induced dissociation (CID) generates fragmentation spectra that can support structure elucidation using chemical interpretation, spectral libraries, or machine learning. Given that metabolites in biological samples are often biosynthetically related, identifying one compound can support annotation of others in the same context. To leverage this, computational strategies such as metabolome networks have been developed, including mass difference networks, spectral similarity networks, and abundance-based correlation networks [15]. These networks group potentially related compounds, aiding annotation, but their biochemical accuracy remains limited. Mass differences may result from multiple structural variations; CID spectral similarity is often low in MS^n^-based data and not always proportional to the structural similarity; and abundance correlations can be misleading due to non-equilibrium conditions or indirect regulation [16,17].

Stable isotope labeling (SIL) provides a complementary chemical approach to resolve these limitations. By tracing the incorporation of heavy atoms (e.g., 13C) from a precursor into downstream metabolites, SIL offers unequivocal evidence of biochemical relationships and can yield structural insights. With modern LC–MS, labeled isotopologues can be comprehensively retrieved using dedicated software [18,19,20,21].

Chicoric acid is a dicaffeoyl tartaric acid ester, recognized for its antioxidant and immunomodulatory properties, that is widely distributed in the plant kingdom. To date, over 25 families have been reported to accumulate chicoric acid, but based on current literature, no reports exist of chicoric acid in flax or other *Linaceae* members. Belonging to the most intensively studied sources, *Echinacea purpurea* (*Asteraceae* family) is currently cultivated on a large scale in China as a medicinal plant and is widely used in drug formulations, nutritional supplements, and health foods [22]. Chicoric acid occurs in different optical isomeric forms, depending on the stereochemistry of the tartaric acid core, but the relationship between the specific isomeric forms and their pharmacological activity remains poorly investigated [22]. The predominant and most commonly reported form is the L-isomer, in which two caffeoyl groups are esterified to L-tartaric acid, as found in *Echinacea* species. In these species, biosynthesis involves a serine carboxypeptidase-like acyltransferase that uses caffeoyl quinate (chlorogenic acid) as the acyl donor and caffeoyl tartaric acid (caftaric acid) as the acyl acceptor [23]. However, this enzymatic activity has not been detected in other L-chicoric acid-producing species of the *Asteraceae* family, such as *Cichorium endivia* and *Lactuca sativa*, suggesting that different species may employ distinct biosynthetic strategies [23]. Moreover, *Equisetum arvense* synthesizes a meso-isomer of chicoric acid via direct acylation of meso-tartaric acid with two caffeoyl moieties from caffeoyl-CoA [24]. These findings collectively point to convergent evolution of chicoric acid biosynthesis, with distinct routes arising independently in different lineages. Further exploring these biosynthetic pathways across diverse species is key to enabling efficient, sustainable production of chicoric acid and its derivatives for pharmaceutical and nutraceutical applications.

In this study, in order to limit the metabolome networks to biochemically relevant conversions, independently of the arbitrarily chosen abundance correlation thresholds or CID spectrum similarity thresholds, we combine the computational approach of mass difference networks with the chemical approach of SIL feeding. After feeding with ^13^C_3_-*p*-coumaric acid, during a time course spanning 24 h, flax seedlings were analyzed at 10 subsequent time points by LC–MS^n^. The study focuses on those metabolites in which the SIL label was detected and which are interconnected by a predefined list of common biochemical conversions. The main phenylpropanoids synthesized in flax seedlings during the studied period were chicoric acid and lariciresinol diglucoside, and sequential waves of transient accumulation of various intermediates, detected by the time-resolved measurements, pointed to potential biosynthesis pathways for these compounds.

## 2. Results

### 2.1. LC–MS Analysis and Tracing SIL Moieties

**Figure 1:**
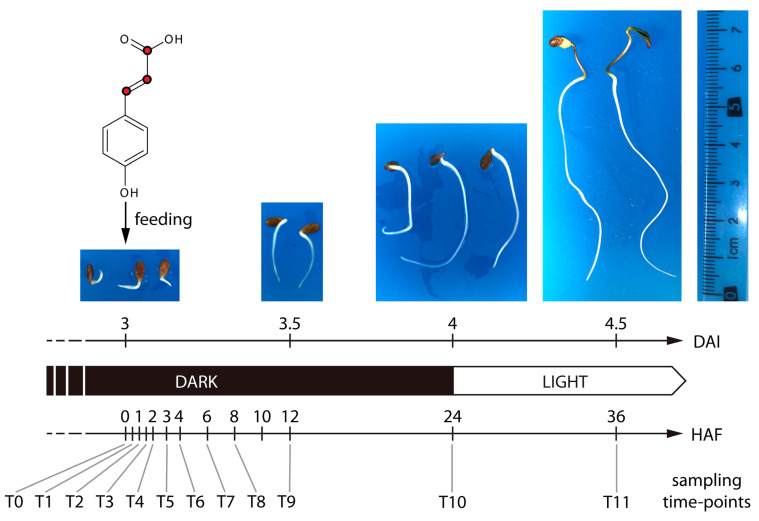
Experimental setup. Three days after the start of imbibition, flax seedlings (*Linum usitatissimum* L.) were fed with [^13^C_3_]-*p*-coumaric acid. The ^13^C-labeled atoms are represented as red dots. Three individual seedlings were sampled at 12 consecutive time points, starting from the time of feeding (T_0_) until 36 h post-feeding (T_11_). DAI, days after imbibition; HAF, hours after feeding.

To study de novo phenylpropanoïd biosynthesis during early developmental stages of flax, we fed 3-day-old dark-grown flax seedlings with a ^13^C_3_-labeled derivative of *p*-coumaric acid, in which the three carbons of the propane side chain were ^13^C-labeled. We sampled flax seedlings directly before feeding (t0), at ten consecutive time points during the 24 h of growth in dark conditions following feeding (t1–t10), and finally 36 h after the start of feeding, corresponding to 12 h after transfer of the seedlings to light conditions (t11) (Figure 1). Methanol extracts of individual seedlings (three replicates per sampling time point) were profiled via reversed-phase HPLC-Ion trap-Orbitrap-MS in negative ionization mode. The processing of all chromatograms resulted in 3487 features, corresponding with an estimated 874 distinct profiled compounds. In the fed samples, metabolites that contain one or more moieties derived from *p*-coumaric acid are expected to be represented by a light feature and one or more heavy features. The light feature is associated with the light isotopologue of the metabolite, arising from the natural, endogenous *p*-coumaric acid precursor. Heavy features are associated with heavy isotopologues of the metabolite, arising from the incorporation of one or more *p*-coumaric acid-1,2,3-^13^C_3_-derived moieties. Within the 874 feature groups estimated to correspond to distinct compounds, we searched for mass differences corresponding to a multiple of the difference between ^12^C_3_ and ^13^C_3_ (∆m/z=3×1.003355 Da), indicating incorporation of a multiple of the three carbon atoms of the propane side chain of *p*-coumaric acid. In total, 86 isotopologue doublets and 17 isotopologue triplets of features differing by (3×1.003355 Da) were detected, corresponding to candidate phenylpropanoid compounds containing one or two *p*-coumaric acid-derived moieties, respectively.

### 2.2. Metabolite Identification Using Candidate Substrate–Product Pairs Network and CID Spectral Interpretation

**Figure 2:**
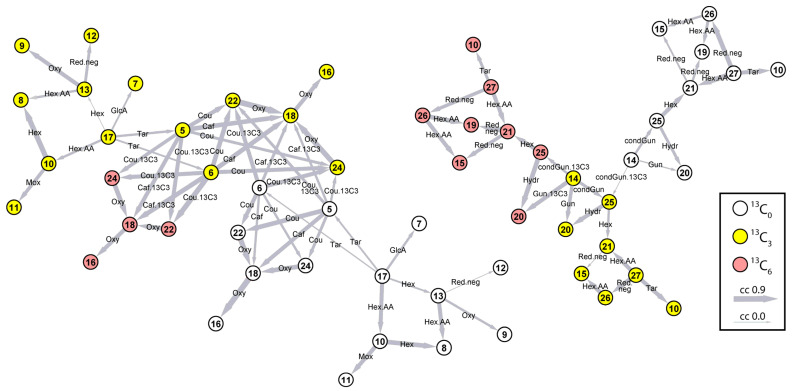
CSPP network. Nodes represent LC–MS features. Node numbers refer to compounds in Table 2. White, yellow, and pink nodes represent native, [^13^C_3_]-, and [^13^C_6_]-labeled isotopologues. Edges represent bioconversions and are labeled with shorthand names defined in Table 1. The edge width reflects the Pearson correlation coefficient between the levels of the CSPP substrates and products (0.0–0.9; all correlations are positive).

All labeled candidate phenylpropanoids retrieved above are potentially biochemically related and may represent substrates and products of enzymatic conversions within a biochemical pathway. Based on the earlier studies of common biochemical relationships among phenolics detected in the LC–MS analyses of Arabidopsis or Maize extracts, we compiled a set of 25 probable biotransformations (Table 1) [16,17]. We then searched for candidate substrate–product pairs (CSPPs) for each of these conversions within the set of 223 LC–MS features belonging to the isotopologue doublets (86 × 2 features) or triplets (17 × 3 features), indicative of the presence of at least one *p*-coumaric acid-derived moiety. A pair of features was declared a CSPP whenever both the *m*/*z* difference and retention time difference between the features corresponded to the mass change and retention time difference expected from the conversion. Out of the 25 targeted conversions, 14 were retrieved at least once among the candidate phenylpropanoid features, and in total 92 CSPPs were defined. The CSPPs were then concatenated into a network in which nodes and edges represent features and conversions (Figure 2). This concatenation resulted in two main networks, involving 57 features, belonging to 11 isotopologue doublets and 9 isotopologue triplets. Four other individual CSPPs were not concatenated to any network. The correlation between the abundances of candidate substrate and product features across all biological replicates was calculated and visualized in the network. The Pearson correlation coefficients ranged from 0.0 (thinnest edges) to 0.9 (thickest edges). Propagating through these two networks, from the simplest compound (*p*-coumaric acid (**17**) and coniferin (**14**), respectively, in the two networks), through subsequent bioconversions, towards increasingly complex compounds, allowed us to annotate the network nodes with candidate structures and to analyze the CID spectra of the most closely related unknown compounds in parallel. Besides, we analyzed the MS^n^ spectra of native, [^13^C_3_]-labeled, and [^13^C_6_]-labeled analogs, represented in the isotopologue doublets or triplets, in parallel (Supplementary Figure S1). A shift of three or two Da observed in product ions or in neutral losses due to labeling could be attributed to the presence of the C_3_ aliphatic side chain of *p*-coumaric acid or only a C_2_ fragment of that aliphatic side chain, respectively. Product ions for which the *m*/*z* values remained unaltered in the MS^n^ spectra of native and [^13^C_3_]-labeled or [^13^C_6_]-labeled analogs, in turn, represented substructures that were not derived from *p*-coumaric acid, such as tartaric acid. This information readily provided structural information about the unknown metabolites and facilitated or corroborated their structural elucidation (Supplementary Figure S1). Compounds were numbered according to their elution order (Table 2). Structural elucidation confidence level ID (compounds **16** and **17**) indicates that compounds were identified by spiking with a reference standard. Compounds annotated with confidence level AN1 are based on accurate mass, MS^n^ spectral elucidation based on well-known gas-phase fragmentations, and, at a minimum, identity confirmation of MS^3^ or MS^4^ spectra, i.e., fragmentation spectra of first-order or higher-order fragments, by comparison with spectra of authentic standards acquired under identical conditions in the same laboratory and stored in an in-house spectral library. These standards included tartaric acid, glucaric acid, or aglycones, such as catechoic acid, hydroxycinnamic acids, and dilignols or lignans. Additional support for level AN1 annotations came from the presence of SIL *p*-coumaric acid-derived signatures in both precursor and fragment ions. Compounds annotated with confidence level AN2 are based on the accurate mass and MS^n^ spectral elucidation based on well-known gas-phase fragmentations (compound **23**), as well as additional evidence from *p*-coumaric acid-derived SIL signatures in the precursor ions and fragment ions (compounds **8**, **10**, **12**, **13**, **18**, **22**, and **24**). For compounds **4, 19**, and **15**, no fragmentation data were available, and these compounds were putatively annotated (PU), based on the accurate mass and the biological context (compound **4**) or based on accurate mass, SIL evidence for the incorporation of two *p*-coumaric acid-derived moieties, and ionization form as acetate adduct, which is characteristic for hexosylated phenolics (compounds **15** and **19**).

The simplest compound retrieved in the first network was the fed tracer *p*-coumaric acid (**17**), present both as a native (grey) or labeled (white) compound. Starting from *p*-coumaric acid, edges representing the addition of a glucose, a glucaric acid, or a tartaric acid moiety led to compounds **5**, **6**, **7**, **10,** and **13**—labeled (white) or unlabeled (grey). These compounds were annotated as coumaroyl tartaric acid isomers (**5**, **6**), coumaroyl glucaric acid (**7**), coumaric acid glucoside (**10**), and coumaroyl glucose (**13**). The *p*-coumaroyl tartaric acid isomers (compounds **5** and **6**), in turn, were connected to higher molecular weight conjugates through the addition of a second *p*-coumaric acid moiety or a caffeic acid moiety, which resulted in compounds **18**, **22**, and **24**, all containing zero, one, or two labeled *p*-coumaric acid-derived moieties (white, yellow, or pink in the network, respectively). These compounds were annotated as dicoumaroyl glucaric acid (**22**, **24**) and coumaroyl-caffeoyl glucaric acid (**18**). Compound **18** was further connected to compound **16** (grey, white, or pink, containing zero, one, or two labeled *p*-coumaric acid-derived moieties, respectively) via an oxygenation edge. Compound **16** was identified as dicaffeoyl glucaric acid or chicoric acid. Glucose conjugates of *p*-coumaric acid (**10** and **13**), on the other hand, were further converted through a single conversion (addition of a second hexose moiety, oxygenation, methoxylation, or reduction) into compounds **8**, **9**, **11,** and **12**, annotated as coumaric acid glucoside glucose ester (**8**), caffeic acid glucoside (**9**), ferulic acid glucoside (**11**), and dihydrocoumaroyl glucose (**12**).

In the second network, only compound **14** was present with zero or one SIL moiety (grey or white). All other compounds in the second network were represented by grey, white, and pink nodes, containing zero, one, or two SIL moieties, respectively. By interpretation of the CID spectra, compound **14** could be annotated as coniferin. Starting from coniferin (**14**), the network was propagated through the addition of a lignin G-unit (**20**) or a condensed G-unit (**25**) and subsequent addition of a hexose moiety (**21**). The MS^n^ spectra confirmed that compounds **20**, **25,** and **21** could be annotated, respectively, as G(8-O-4)G glucoside, dehydrodiconiferyl alcohol glucoside (DDC glucoside, G(8-5)G hexoside), and pinoresinol diglucoside. Pinoresinol diglucoside (**21**) was further converted by reductions into compounds **15** and **19**, putatively annotated as two lariciresinol diglucoside isomers. Finally, pinoresinol diglucoside (**21**) and lariciresinol diglucoside (**15** and **19**) were connected via the loss of a hexose to compounds **27** and **26**, respectively, annotated as pinoresinol glucoside and lariciresinol glucoside.

Overall, all nodes in the two networks could be structurally characterized and represented in a total of 22 different phenolic compounds. The first network was entirely composed of hydroxycinnamic acid ester and glucoside derivatives. The second network consisted of glucosylated coniferyl alcohol (coniferin) and coniferyl alcohol-derived dilignols or lignans.

Besides the compounds connected in the CSPP networks, other phenolics were characterized that could not be detected by the procedure described above. These comprised caffeoyl tartaric acid (**1**), which was only detected as a native compound and not under the labeled form, as well as the C_6_C_1_ phenolics protocatechoyl glucose (**2**), *p*-hydroxybenzoic acid glucoside (4HBA glc) (**3**), gallic acid glucoside (**4**), and the C_6_C_2_ phenolic hydroxyphenyl acetic acid (**23**). A total of 27 different structures were thus elucidated (Table 2). The ^13^C_1_ isotopologue was detected for protocatechoyl glucose (**2**) and *p*-hydroxybenzoic acid glucoside (**3**) but was under the detection limit for gallic acid glucoside (**4**), indicating that the latter compound was not derived from the exogenously administered SIL *p*-coumaric acid. For hydroxyphenyl acetic acid (C_6_C_2_) (**23**), the ^13^C_1_ isotopologue was detected, but not the ^13^C_2_ isotopologue. Further analysis of the enrichment of the ^13^C_1_ isotopologues of compounds **2**, **3**, and **23** is conducted below. For all compounds listed in Table 2, MS^n^ spectra are available in Supplementary Table S1, and mass spectral interpretations are described in Supplementary Methods S1.

### 2.3. Correlation and Time-Course Clustering

**Figure 3:**
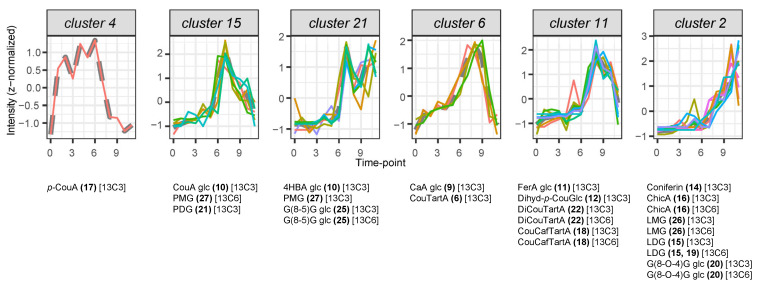
Hierarchical clustering of heavy isotopologue time-course profiles. Time-course profiles were constructed using average intensity values across three biological replicates for each feature at each time point (t). Distinct colors are used to differentiate features within each graph. Note that color assignments are independent across graphs; therefore, identical colors in different graphs do not indicate the same feature. Values are z-normalized before clustering. Compound name abbreviations and numbers refer to compound full names listed in Table 2. Multiple clustered features per mentioned compound result from alternative ionization forms, such as dimers. Cluster numbers correspond to clusters in Supplementary Figure S2. Time points 0 to 11 correspond to the sampling times as follows: immediately before feeding (72 h after imbibition, time point 0) and at 0.5, 1, 1.5, 2, 3, 4, 6, 8, 12, 24, and 36 h after the start of feeding with *p*-coumaric acid-1,2,3-^13^C_3_ (time points 1 to 11).

To investigate the biochemical relevance of the CSPPs in the biosynthesis pathways actually taking place in the studied flax seedlings, we examined temporal correlations in compound abundance. We generated time-course profiles for all light and heavy features of the 27 structurally characterized compounds by calculating the average intensity across three biological replicates at each time point. The light features comprised the ^12^C isotopologues, derived from endogenous metabolism. The heavy features comprised the ^13^C_3_ and ^13^C_6_ isotopologues of the compounds represented in the network (Figure 2), as well as the ^13^C_1_ isotopologue of compounds **2**, **3**, and **23**, potentially partially derived from exogenously administered ^13^C_3_-*p*-coumaric acid. Furthermore, features corresponding to alternative ionization forms of the same compounds, such as dimers [2M−H], were included in the time-course clustering. Correlations among these time-course profiles were calculated and visualized through cluster analysis (Supplementary Figure S2). Hierarchical clustering resulted in a cluster comprising both light and heavy features of protocatechoyl glucose (cluster 29), a cluster grouping the light and heavy features of hydroxyphenyl acetic acid (cluster 7), six clusters grouping exclusively light features, six clusters grouping exclusively heavy features, one cluster grouping heavy features as well as both light and heavy features of coumaric acid glucoside glucose ester (**8**), and 15 individual features that were not correlated in time with any other feature. The observation that the abundance of light and heavy isotopologues was correlated over time for protocatechoyl glucose (**2**), hydroxyphenyl acetic acid (**23**), and coumaric acid glucoside glucose ester (**8**) suggested that both isotopologues were entirely native and that these compounds were not derived from the fed ^13^C_3_-*p*-coumaric acid during the studied time course. The differential time-course profiles between light and heavy isotopologues for all other compounds, in turn, indicate that the heavy isotopologues of these compounds, including for *p*-hydroxybenzoic acid (**3**), were derived from the fed ^13^C_3_-*p*-coumaric acid. The accumulation of heavy ^13^C_3_ *p*-coumaric acid did not cluster together with any other metabolite. A strong accumulation of heavy *p*-coumaric acid remained stable from 30 min (first time point after feeding) till four hours after the start of feeding, after which its accumulation declined rapidly (cluster 4). Clusters 15, 21, 6, 11, and 2 showed successive transient waves of accumulation of distinct heavy features, with maximal abundance at time points going from 4–6 h (cluster 15) to 36 h (cluster 2) after the start of the time course (Figure 3). Metabolization of SIL *p*-coumaric acid started four hours after the start of feeding, as could be deducted from its rapid decrease, concomitant with the appearance of SIL *p*-coumaric acid glucoside, SIL *p*-hydroxybenzoic acid glucoside, and hexosylated or dihexosylated SIL lignans pinoresinol and dehydrodiconiferyl alcohol (DDC) (clusters 15 and 21). Shortly after this, around 6 h to 8 h after the start of feeding, a transient appearance of hydroxycinnamic acid glucosides and conjugates with tartaric acid was observed (clusters 6 and 11). The end of the time course was characterized by the appearance of SIL coniferin, SIL G(8-O-4)G hexoside, SIL lariciresinol mono- and diglucoside, and SIL chicoric acid (cluster 2). In cluster 2, SIL G(8-O-4)G hexoside levels peaked at 24 h after feeding (t10), whereas the accumulation of other SIL compounds continued increasing till the end of the time course. Also in cluster 2, one compound showed a clear additional early peak at 3 h after feeding (t5)—this compound corresponds to coniferin.

**Figure 4:**
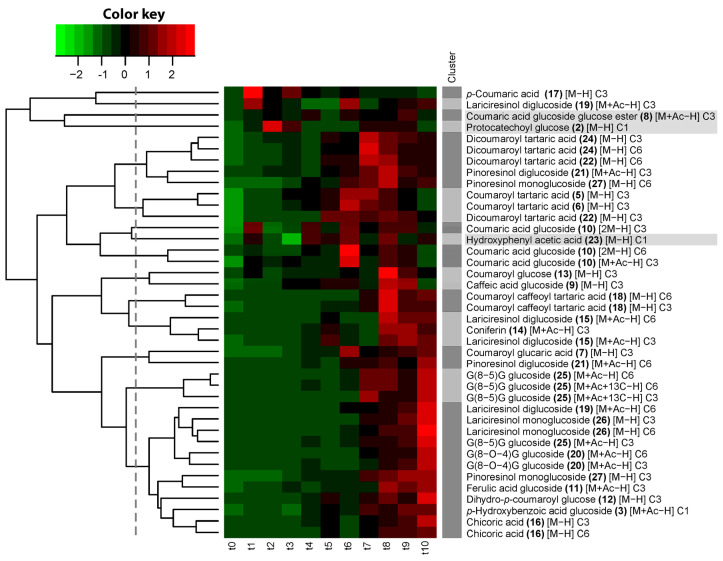
Hierarchical clustering of heavy to light isotopologue ratios of structurally characterized compounds accumulating in flax seedlings (*Linum usitatissimum* L.) during 24 h period after feeding with *p*-coumaric acid-1,2,3-^13^C_3_. Time point t0 corresponds to the sampling time immediately before feeding (72 h after imbibition). Time points t1 to t10 correspond to the sampling times at 0.5, 1, 1.5, 2, 3, 4, 6, 8, 12, and 24 h after the start of feeding. Dark grey and light grey shaded clusters are defined by the cut-off of the dendrogram, designated by the vertical dashed line. Compound numbers refer to compounds listed in Table 2. Grey shaded compounds are not ^13^C-enriched. Text between square brackets refers to ionization form: [M−H], molecular ion; [2M−H], dimer; [M+Ac−H], acetate adduct; […+13C], isotopologue with one extra natural ^13^C. C6, C3, and C1 refer to ratios of ^13^C_6_, ^13^C_3_, and ^13^C_1_ isotopologues over the corresponding monoisotopic ^12^C isotopologue.

To follow the incorporation of SIL moieties from ^13^C_3_ *p*-coumaric acid into the compounds, we performed a time-course analysis of the ratios of each heavy feature (^13^C_6_, ^13^C_3_, or ^13^C_1_) to its corresponding native feature, followed by a hierarchical clustering of these incorporation time courses. The entirely native nature of compounds **2**, **8**, and **23** was further corroborated by the observation that the heavy/light ratio profiles of compounds **2** and **8** clustered together and that, for compound **23**, the heavy/light ratio was not minimal at the time point before feeding, indicating that the heavy feature of this compound was not derived from ^13^C_3_ *p*-coumaric acid. The cut-off defining the threshold for significant clustering was therefore set at the dendrogram height where the presumably fully native compounds **2**, **8**, and **23** no longer clustered with SIL-labeled compounds (Figure 4). The ratio of heavy to light *p*-coumaric acid was maximal 30 min after the start of feeding (t1) and decreased rapidly afterwards. For other compounds, the incorporation of SIL reached a maximum at later time points. Successive maxima of SIL enrichment were observed for different compounds in the following order: first coumaric acid glucoside (maximum at t6), then coumaroyl tartaric acid and dicoumaroyl tartaric acid (enrichment in ^13^C_6_ for the two isomers and enrichment in ^13^C_3_ for one isomer) (maximum at t6–t7), then dicoumaroyl tartaric acid (enrichment in ^13^C_3_ for the second isomer) and pinoresinol monoglucoside (maximum at t7–t8), then G(8-5)G hexoside (enrichment in ^13^C_6_ for the two isomers and enrichment in ^13^C_3_ for one isomer, maximum at t7–t10), then coumaroyl glucose and caffeic acid glucoside (t8–t9), then coumaroyl caffeoyl tartaric acid (maximum at t8 and slightly decreasing afterwards), then coniferin and lariciresinol diglucoside (two maxima: early maximum at t5 and late maximum at t8–t10), and finally a large group containing a second lariciresinol diglucoside isomer, G(8-5)G hexoside (enrichment in ^13^C_3_ for the second isomer), lariciresinol monoglucoside, ferulic acid glucoside, dihydrocoumaroyl glucose, *p*-hydroxybenzoic acid glucoside, G(8-O-4)G hexoside, and chicoric acid (maximum at t10).

For the caffeoyl tartaric acid conjugates, caffeoyl coumaroyl tartaric acid and dicaffeoyl tartaric acid (chicoric acid), incorporation of a single SIL moiety was detectable immediately after the start of feeding, whereas the incorporation of two SIL moieties appeared only after a delay. The delay for the appearance of ^13^C_6_-labeled chicoric acid was shorter than that of ^13^C_6_-labeled coumaroyl caffeoyl tartaric acid (t5 and t7, respectively), but it should be noted here that, in absolute terms, the intensity of chicoric acid signals was one to two orders of magnitude larger than coumaroyl caffeoyl tartaric acid signals, so it can be expected that lower abundant isotopologues remain longer under the detection limit for the latter.

### 2.4. Quantification of Chicoric Acid

**Figure 5:**
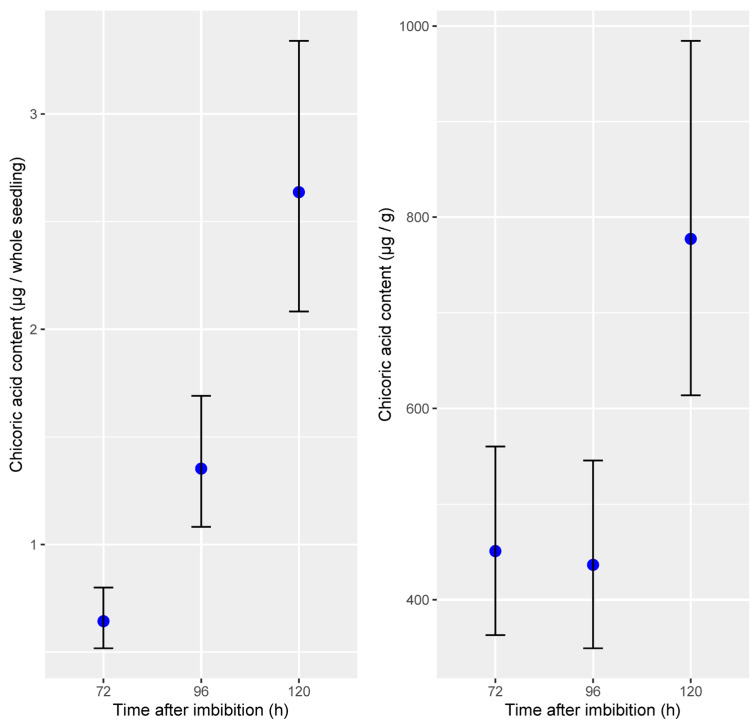
Absolute chicoric acid quantification in flax sprouts (*Linum usitatissimum* L.). Blue dots and error bars represent mean predicted content and 95% confidence intervals (*n* = 5).

Based on a dilution series of an authentic L-chicoric acid standard, we quantified chicoric acid content in flax sprouts that were cultivated in identical conditions as used for the feeding experiments and harvested 72, 96, and 120 HAI, corresponding to 3-, 4-, and 5-day-old flax sprouts. Chicoric acid content per seedling comprised between 0.5 and 0.8 µg, between 1.1 and 1.7 µg, and between 2.1 and 3.3 µg at 72, 96, and 120 HAI, respectively, based on 95% confidence intervals. When normalized to dry tissue weight, however, the chicoric acid content remained stable in 3- and 4-day-old flax sprouts (350–560 µg/g) and reached levels between 600 and 1000 µg in 5-day-old flax sprouts (Figure 5).

## 3. Discussion

### 3.1. SIL, MS^n^, and CSPP: A Synergistic Combination

Spectral similarity is often an essential parameter in the construction of networks of structurally related compounds, which in turn can greatly facilitate structural elucidation. The GNPS algorithms annotate pairs of LC–MS features based on CID spectral similarity first and then compute the mass difference between neighboring features. The CSPP algorithm offers an alternative that first connects a pair of features whenever the mass difference and the elution order correspond to those expected for known biochemical reactions [17]. False conversions are removed a posteriori based on a threshold of spectral similarity or abundance correlation [17]. Both approaches depend, therefore, strongly on the method used to compute the spectral similarity and on the type of mass spectra that were generated, and both approaches will generally fail to annotate biochemically related features if structural similarity was not reflected in a similar fragmentation, for example, due to the influence of a distinct functional group or due to a low number of product ions, which is often the case for ion-trap-generated multistage MS^n^ spectra.

In this study, feeding flax seedlings with SIl *p*-coumaric acid allowed us to preselect *p*-coumaric acid-derived compounds, based on the detection of isotopologue multiplets, for the construction of a CSPP network. The detection of at least one labeled analog for all nodes of the network certifies their biochemical relationship, so that no further curation of the network via abundance correlation or spectral similarity was needed. The large range of correlation coefficients, going from 0.0 to 0.9, observed among biochemically related compounds in the CSPP network in this study, illustrates well the risk of using abundance correlation as a parameter for biochemical relationships. Inspection of the MS^n^ spectra of the compounds in the CSPP network illustrates that true structural similarity often could not have been revealed based on counting the number of common product ions or neutral losses and/or dot product computations. For example, the highly similar structures and fragmentation pathways of dicoumaroyl tartaric acid and chicoric acid did not result in any common product ion or common neutral loss in their MS^2^ spectrum (Supplementary Figure S1). This observation also highlights the importance of developing more complex structural similarity computations, taking fragmentation paths and interdependence of fragments into account.

Although they do not necessarily reflect true steps in a biosynthesis pathway, almost all connections in the CSPP network represented valid biochemical conversions. For example, a rare exception was observed in the connection between a homodimer adduct of *p*-coumaric acid glucoside and the pseudomolecular ion of pinoresinol monoglucoside due to an *m*/*z* difference that corresponded fortuitously to the addition of tartaric acid. This example emphasizes the importance of detecting and treating homo- and hetero-adducts among the LC–MS features prior to the construction of CSPP networks if more complex datasets are analyzed. The multiplicity of the isotopologues of the compounds in the network revealed the number of *p*-coumaric acid-derived moieties, and comparative multiple-stage MS^n^ for native and differentially labeled isotopologues of the same compound allowed tracing SIL moieties in the different fragmentation pathways and therefore unambiguously assigning *p*-coumaric acid-derived and non-derived substructures to the main first-order product ions of each fragmented compound. All nodes in the CSPP network could as such be structurally elucidated, revealing that the phenolic profile of three-to-four-day-old flax seedlings was composed of hydroxycinnamic acid hexosides or conjugates, hexosylated (neo)lignans/dilignols, and benzenoids. Therefore, SIL feeding analysis, together with the CSPP approach and multiple-stage MS^n^ fragmentation, forms a synergistic combination, facilitating the grouping and structural elucidation of biochemically related compounds, and the approach is highly promising for application in the analyses of larger metabolomic studies covering more metabolites after longer periods of SIL feeding.

### 3.2. Divergent Pathways of Coniferyl Alcohol Utilization During Flax Germination

**Figure 6:**
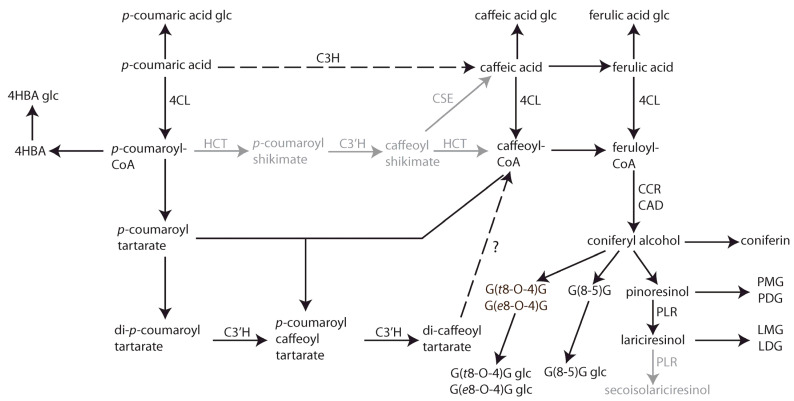
Phenylpropanoid biosynthesis pathways deducted from SIL feeding and time-course experiment in flax seedlings (*Linum usitatissimum* L.). Dashed arrows indicate two potential fluxes from *p*-coumaric acid to coniferyl alcohol and derivatives. Compounds in grey were not observed in this study. Metabolites: 4HBA, *p*-hydroxybenzoic acid; LDG, lariciresinol monoglucoside; LMG, lariciresinol monoglucoside; PDG, pinoresinol diglucoside; PMG, pinoresinol monoglucoside. Enzymes: 4CL, 4-coumaroyl-CoA ligase; C3H, coumarate 3-hydroxylase; C3′H, coumaroyl shikimate/quinate 3′-hydroxylase; CCR, cinnamoyl-CoA reductase; CAD, cinnamyl alcohol dehydrogenase; CSE, caffeoyl shikimate esterase; HCT, hydroxycinnamoyl-CoA:shikimate/quinate hydroxycinnamoyltransferase; PLR, pinoresinol lariciresinol reductase.

The CSPP network, in combination with a time-course analysis with high temporal resolution, allowed us to investigate hydroxycinnamic acid, benzenoid, and (neo)lignan and/or lignin biosynthesis during flax seedling development (Figure 6). Focusing first on (neo)lignans, the coniferyl alcohol coupling products DDC, pinoresinol, and lariciresinol were present only as single stereoisomers, in accordance with the involvement of a dirigent protein, and they accumulated under hexosylated or dihexosylated form, supporting their identification as lignans and not as lignin precursors. Biosynthesis of pinoresinol and DDC from the fed precursor started without delay, immediately concomitant with the appearance of SIL coumaric acid glucoside. Whereas the levels of SIL DDC hexoside remained elevated for the remainder of the time course, SIL pinoresinol mono- or dihexoside accumulation was transient and was followed by the rise in the levels of SIL lariciresinol mono- or dihexoside. This observation reflects the activity of a pinoresinol lariciresinol reductase (PLR) enzyme. In flax, two PLR enzymes with opposite enantiospecificity are cloned: PLR-LU1 and PLR-LU2. In vitro PLR enzymatic activity assays have shown that PLR-LU1 reduces RR-pinoresinol directly to RR-secoisolariciresinol, without intermediate accumulation of lariciresinol. PLR-LU2 converts SS-pinoresinol first to SS-lariciresinol and then further reduces the accumulating lariciresinol to SS-secoisolariciresinol [25]. Our findings suggest the activity of PLR-LU2 or of an enzyme with similar activity in flax seedlings. Strictly speaking, pinoresinol and lariciresinol are lignans, and DDC is a neolignan, because of the difference in bond (8-8 bond versus 8-5 bond). In contrast, the detected SIL G(*t*8-O-4)G and G(*e*8-O-4)G hexosides resembled hexosylated dilignol lignin precursors because both *erythro* and *threo* stereoisomers were formed in equivalent amounts, in accordance with unguided combinatorial coupling. Moreover, the accumulation of the presumed lignin precursors was not coordinated with the accumulation of SIL lignans. Nevertheless, no other higher-order SIL oligolignols were detected, and no unhexosylated oligolignols were detected, indicating that proper lignification had not started in the flax seedlings during the time window of the study. The accumulation of the SIL G(*t*8-O-4)G and G(*e*8-O-4)G hexosides increased relatively late but peaked at the penultimate time point, after which it decreased again. This could indicate that, after temporary accumulation under the hexosylated form, the G(*t*8-O-4)G and G(*e*8-O-4)G aglycones were released in the four-day-old seedlings and were incorporated into lignin or elongated to higher-order oligolignols.

All lignans, neolignans, and dilignols observed were derived from coniferyl alcohol. SIL coniferyl alcohol accumulated under the hexosylated form as coniferin, first transiently, very early in the experiment, immediately preceding the accumulation of the lignans DDC and pinoresinol, and then again and more strongly at the end of the time course, concomitant with the decrease in SIL ferulic acid glucoside levels. This pattern reflects two independent fluxes toward coniferyl alcohol. The fast flux seems to serve the production of the lignans DDC and pinoresinol. The velocity of this flux, the absence of the accumulation of intermediates, and the absence of G(*t*8-O-4)G and G(*e*8-O-4)G combinatorial coupling products concomitantly with the lignans suggest metabolic channeling from *p*-coumaric acid all the way to the lignans, mediated by enzyme interaction or colocalization. The slower flux toward coniferyl alcohol, for which no evidence of enzymatic channeling was observed, probably mainly feeds early lignification in the flax seedlings, starting from the end of the time frame of this experiment.

### 3.3. Possible Alternative 3′-Hydroxylation Pathways in Flax Seedlings

The high temporal resolution of the experiment enabled us to track successive waves of intermediate accumulation, provided the pathway is not channeled and intermediates diffuse freely between enzymatic steps. For example, two hours after the peak of SIL *p*-coumaric acid glucoside accumulation, SIL caffeic acid glucoside peaked, and again four hours later, SIL ferulic acid glucoside accumulation peaked, reflecting successive 3-hydroxylation and 3-O-methylation of the aglycone. Moreover, the decrease in SIL ferulic acid glucoside levels coincided with the late SIL coniferin peak, suggesting a metabolic flux from ferulic acid toward coniferyl alcohol and its glycosylated form.

The introduction of hydroxyl groups in the meta-position of *p-*coumaric acid-derived esters is known to be catalyzed by cytochromes P450 monooxygenases from the CYP98 family [26,27,28]. The majority of CYP98s with identified activities preferentially use *p*-coumaroyl shikimate or *p*-coumaroyl quinate as substrates and show minor activity on free *p*-coumaric acid [29,30]. For the formation of lignin G and S monomers, the CoA-activated 4-coumaroyl moiety first needs to be esterified with shikimate or quinate by hydroxycinnamoyl-CoA:shikimate/quinate hydroxycinnamoyltransferase (HCT), after which CYP98 members catalyze the 3′-hydroxylation of *p*-coumarate esters [2,31]. The caffeic acid moiety can then be retransferred back to CoA by HCT or released by CSE to serve further biosynthetic purposes [32]. The enzymes HCT and *p*-coumaroyl shikimate/quinate 3′-hydroxylase (C3′H) are considered as the entry points of the phenylpropanoid pathway to lignification and are highly conserved among all land plants [31]. In 4-day-old etiolated flax seedlings, the expression of putative homologues of *Arabidopsis* genes encoding a CYP98 member and an HCT, along with other phenylpropanoid biosynthesis enzymes, including PAL, C4H, COMT, CCoAOMT, CCR, and CAD, has been previously reported [33]. Therefore, it is remarkable that, in the present study, no shikimate or quinate esters of *p*-coumaric or caffeic acid were detected. Instead, we detected SIL *p*-coumaroyl glucose, SIL *p*-coumaroyl glucarate, and SIL *p*-coumaroyl tartarate esters. The successive transient accumulation of SIL *p*-coumaroyl tartarate, SIL dicoumaroyl tartarate, and SIL caffeoyl coumaroyl tartarate, followed by the rise in SIL dicoumaroyl tartarate (chicoric acid) levels, suggests two successive 3′hydroxylations of dicoumaroyl tartarate to form chicoric acid, via a *p*-coumaroyl caffeoyl tartarate intermediate, in flax seedlings. The first step could also be achieved without involving 3′hydroxylation at the level of the tartarate ester, as the *p*-coumaroyl caffeoyl tartarate intermediate could also be the product of caffeoyl transfer from caffeoyl-CoA to *p*-coumaroyl tartarate. Indeed, SIL caffeic acid glucoside transiently accumulated before the appearance of SIl *p*-coumaroyl caffeoyl tartarate (Figure 3, clusters 6 and 11), indicating that the 3′hydroxylation leading to caffeic acid moieties occurred, at least partially, independently from the tartarate ester. However, the absence of SIL caffeoyl tartarate in the present study leaves *p*-coumaroyl caffeoyl tartarate as the only and best candidate precursor of chicoric acid. This latter reaction implies a 3′hydroxylation at the level of the tartaric acid ester.

The question remains if the caffeoyl moieties of chicoric acid contribute to coniferyl alcohol and lignin biosynthesis in early developmental stages of flax seedlings. Our data do not support this hypothesis. The proportion of labeled chicoric acid is still on the rise at the last time point of the study, after transfer of the seedlings to light exposure, whereas for coniferin, the proportion of labeled isotopologue reached a maximum more than 24 h earlier. If SIL coniferin was a product of SIL chicoric acid, such an early drop in the proportion of SIL coniferin could not be easily explained. It seems, therefore, more plausible that the 3-hydroxylation leading to coniferyl alcohol and oligolignols in flax seedlings occurred at the level of free *p*-coumaric acid, independently from the tartaric acid conjugates.

### 3.4. Chicoric Acid

This study demonstrates that flax seedlings accumulate L-chicoric acid—the same stereoisomer recognized for its pharmacological properties and typically extracted from *Echinacea* and *Cichorium intybus* (chicory). Chicoric acid content in flax sprouts could, besides lignans, considerably contribute to the value of flax sprouts as functional food. Chicoric acid was first isolated from chicory leaves but has since been identified in a wide range of plant species, including seagrass, horsetail, ferns, lettuce, and basil [34]. These dicaffeoyl esters are proposed to serve as antioxidant reservoirs within plant tissues. In several species, including *Vitis vinifera* and artichoke, the accumulation of di-caffeoyl quinic acids increases upon UV exposure [35,36]. Similarly, in developing flax seedlings, the accumulation of chicoric acid likely functions as a protective mechanism against initial light and UV exposure. Supporting this hypothesis, chicoric acid levels remained stable in seedlings kept in darkness (3- and 4-day-old) but increased significantly after transfer to light. The levels of chicoric acid detected in 5-day-old flax seedlings transferred to light after 4 days of germination were comparable to the lower end of the range reported for the aerial parts of *Cichorium intybus*, for which concentrations ranging from 90 to 8300 µg/g are reported, depending on the study [22,37,38]. The concentrations of chicoric acid reported in plants are typically highly variable and are probably influenced by factors such as growth conditions, harvest timing, and post-harvest processing. This high variability suggests a potential for enhancing chicoric acid accumulation in flax sprouts through optimized cultivation practices and a better understanding of its biosynthetic pathway.

In the present study, none of the known precursors of chicoric acid biosynthesis in *Echinacea*, such as caffeoyl tartarate or chlorogenic acid, were detected among the labeled (SIL) compounds. Caffeoyl tartarate could be detected only in its native (unlabeled) form, indicating that it is unlikely to serve as a biosynthetic precursor in our experimental context—particularly since chicoric acid was observed with two labeled caffeoyl moieties. Instead, the coordinated disappearance of SIL *p*-coumaroyl caffeoyl tartarate and SIL di-*p*-coumaroyl tartarate, concomitant with the rise in SIL chicoric acid levels, strongly suggests an alternative route in flax seedlings: one or two meta-hydroxylations on the *p*-coumaroyl ring(s) of these esters likely yield chicoric acid.

Although no meta-hydroxylation activity has yet been reported on *p*-coumaroyl tartarate esters in the context of chicoric acid biosynthesis, such enzymatic functions are well documented in other phenylpropanoid pathways. In dicots, several HCT and C3′H paralogs have evolved functions beyond lignification, contributing to the biosynthesis of soluble phenolic esters and amides [31]. A major duplication event in HCT gave rise to a clade of quinate-acylating enzymes responsible for chlorogenic acid biosynthesis [39]. Likewise, Arabidopsis CYP98s can hydroxylate tricoumaroylspermidine to tricaffeoylspermidine [40], and several CYP98 enzymes from *Lamiaceae* and *Boraginaceae* catalyze the meta-hydroxylations of 4-coumaroyl-(R)-3-(4-hydroxyphenyl)lactate to produce rosmarinic acid [40,41,42]. These precedents support the plausibility of a yet-uncharacterized CYP98-dependent route converting coumaroyl tartarate esters into chicoric acid in flax.

### 3.5. Benzoic Acids

Plant benzoic acids can be derived from intermediates of the shikimate/chorismate pathway, upstream of phenylalanine, or they can be derived from cinnamic acid and its hydroxy- and methoxy-derivatives, through shortening of the C_3_ precursor side chain by two carbon units. In the present feeding experiment with stable isotope-labeled (^13^C_3_)*p*-coumaric acid, catechuic and gallic acid were not enriched in ^13^C_1_ in the (^13^C_3_)*p*-coumaric acid-fed flax seedlings, in accordance with these compounds being derived from the upstream shikimate pathway. In contrast, 4HBA was actively derived by chain shortening from the fed (^13^C_3_)*p*-coumaric acid, as evidenced by the accumulation profile of (^13^C_1_)4HBA and the increasing ^13^C_1_/^13^C_0_ isotopic ratios. Multiple pathways have been proposed for this side chain shortening reaction: the CoA-dependent β-oxidative route, the CoA-dependent non-oxidative route, and the CoA-independent non-oxidative route [43]. The characteristic feature of the non-oxidative pathways is the presence of benzaldehydes as key metabolic intermediates in the formation of benzoic acids. The absence of labeled or unlabeled 4-hydroxybenzaldehyde supported a β-oxidative route for the biosynthesis of 4HBA in flax seedlings.

## 4. Materials and Methods

### 4.1. Chemical Products

Stable isotopically labeled *p*-coumaric acid-1,2,3-^13^C_3_ and an authentic chicoric acid standard were purchased from Sigma-Aldrich (St. Louis, MO, USA).

### 4.2. Plant Growth, Feeding, and Harvesting Conditions

Flaxseeds (*Linum usitatissimum* L. ‘Baladin’) cultivated in 2012 were kindly provided by Laboulet (Airaines, France), a certified supplier of agricultural seed varieties. The identity of the plant material was based on the supplier’s documentation and certification, in accordance with EU seed marketing standards. No further morphological or molecular authentication was performed, and no voucher specimen was deposited. Aliquots of 1.2 g of seeds were disinfected for 15 min in 7 mL methanol and, after disinfection, imbibed for 5 min in 10 mL sterile distilled water. Immediately after imbibition, seeds were placed on glass Petri dishes (15 cm diameter, 30 seeds per dish) containing 15 mL of 10-fold diluted sterile liquid Murashige–Skoog (MS) medium (Duchefa, Haarlem, The Netherlands) and incubated in a dark culture room at 23 °C. Four days (96 h) after the start of imbibition (24 h after the start of feeding), the Petri dishes with flax seedlings were transferred to a light culture room (photoperiod 16 h; 3350–3500 lux, 23 °C). For the SIL feeding experiment, three days (72 h) after the start of imbibition, germinated seeds were transferred to Petri dishes containing 15 mL of 0.5 mM *p*-coumaric acid-1,2,3-^13^C_3_ in 10-fold diluted sterile liquid MS medium and further incubated at 23 °C in the dark room. At 72 HAI, directly before feeding (T0), and at 0.5 h, 1 h, 1.5 h, 2 h, 3 h, 4 h, 6 h, 8 h, 12 h, 24 h, and 36 h after the start of feeding with *p*-coumaric acid-1,2,3-^13^C_3_, three germinated seeds were individually directly frozen in liquid nitrogen and freeze-dried for two days. For the absolute quantification of chicoric acid, no feeding was performed, and five individual seedlings were harvested at 72 h, 96 h, and 120 h (3, 4, and 5 days) after the start of imbibition.

### 4.3. Metabolite Extraction

The individual seedlings were homogenized with a Mixer Mill MM400 (Retsch GmbH, Haan, Germany) for 1 min at 30 Hz and extracted with 500 µL of methanol for 10 min at 50 °C, agitating at 2000 rpm in an Eppendorf thermomixer (Eppendorf, Hamburg, Germany). Following evaporation of the methanol supernatant, the pellet was dissolved in 1 mL of milliQ water/cyclohexane (Sigma Aldrich, Saint Louis, MO, USA) (1/1, *v*/*v*); the tubes were then vortexed and centrifuged at 14,000 rpm (20,000 g) for 10 min. Finally, 200 μL of the lower aqueous phase was transferred to an LC vial and used for LC–MS analysis.

### 4.4. Quantification of Chicoric Acid

A dilution series of authentic chicoric acid was prepared at 20 µM, 10 µM, 5 µM, 2 µM, 1 µM, 0.5 µM, 0.2 µM, 0.1 µM, 0.05 µM, and 0.01 µM. LC–MS analyses of extracts of unfed seedlings (five biological replicates per developmental stage, 3 developmental stages) and authentic chicoric acid dilutions (three technical replicates per dilution) were performed on an ACQUITY UPLC I-Class system coupled to a Vion IMS QTof (Ion Mobility Quadrupole Time-of-flight) hybrid mass spectrometer, equipped with an electrospray ionization (ESI) source (Waters, Manchester, UK), as previously described [44]. Ionization was set in the positive mode. Data acquisition and peak integration were performed with UNIFI software (V1.9.2, Waters). Peak area integration was performed in an EIC defined at *m*/*z* 497.06905 [chicoric acid +Na]^+^, with a tolerance of 5 ppm. A calibration curve (R-squared: 0.98) was created by linear regression in R version 4.4.1 [45]. Point estimates and 95% confidence intervals of chicoric acid concentrations were predicted using the inverse.predict function of the chemCal R package version 0.2.3 [46]. Dry weight of individual extracted seedlings was measured after methanol extraction. Predicted molar concentrations were back-transformed to be expressed in terms of µg per seedling and µg per g dry extracted seedling tissue.

### 4.5. Untargeted LC–MS Analysis of SIL Flax Seedlings

LC–MS analyses of extracts of *p*-coumaric acid-1,2,3-^13^C_3_-fed seedlings were performed on a Dionex Ultimate module 3000 LC system (Thermo Scientific Dionex, Sunnyvale, CA, USA), connected to an LTQ Orbitrap XL hybrid FTMS (Thermo Fisher Scientific, Waltham, MA, USA) via an electrospray source (ESI) in negative ionization mode. A reversed phase separation was performed using a Sunfire C18 column (150 × 9 × 3 mm, 3.5 µm; Waters, Milford, MA, USA). All LC–MS conditions were as previously described [4]. Full Fourier transform-MS spectra were recorded between 120 and 2000 *m*/*z*. Data-dependent MS^n^ spectra were recorded on the ion trap MS using the preliminary low-resolution data obtained during the first 0.1 sec of the previous full Fourier transform-mass spectrometry scan: an MS2 scan of the most abundant *m*/*z* ion of the full Fourier transform-mass spectrometry scan, followed by MS3 scans of the two most abundant first-order product ions and an MS4 scan of the most abundant second-order production. MSn scans were obtained with 35% collision energy. Peak finding, peak integration, and retention time correction were performed using the xcms R package version 1.38.0 [47] after conversion of the original RAW files to an open-source format ‘mzXML’ with the MSconvert tool from ProteoWizard version 3.0.5163 [48]. Grouping of LCMS features estimated to belong to the same compounds was performed using the CAMERA R package version 1.64.0 [49]. User-defined parameters used for LC–MS data processing are detailed in Supplementary Methods S2. Chemical formulae of compounds were obtained in Xcalibur version 1.2 (Thermo Fisher Scientific, Waltham, MA, USA), and the same program was used to analyze the MS^n^ spectra.

### 4.6. Tracing Labeled Metabolites

Tracing the complete set of labeled metabolites in an unbiased manner occurred via two functions of the CAMERA R package version 1.64.0 [49]. First, features eluting within the same retention time window were grouped using the groupFWHM function. Within these feature groups, we traced doublets or series of isotopologues, carrying zero to five ^13^C_3_ SIL moieties, using the findIsotopes function, in which we replaced the default isotopeMatrix by a custom *m*/*z*-difference and ratio matrix. The minimum and maximum *m*/*z* differences for matching isotopic peaks were set at 3, 6, 9, 12, or 15 times 1.003355 Da ± 0.005 Da (Supplementary Methods S2).

### 4.7. Mass Difference Networks

A ruleset of mass differences and retention time differences was defined for a list of conversions. For those biotransformations involving the addition of phenylpropanoid moieties, such as the addition of coumaric acid, ferulic acid, sinapic acid, or of a guaiacyl moiety, we also added the SIL derivatives of the biotransformations to the list. The mass differences were defined with four decimals. The sign of each mass difference was determined by the difference in retention time between substrate and product: positive if the product is expected to have a larger retention time than the substrate (for example, a feruloylation leads to a more apolar product, with a higher retention time), but negative if the product is expected to have a shorter retention time than the substrate (for example, a hydroxylation leads to a more polar substrate, with a shorter retention time). Mass differences between LC–MS features were calculated as *m*/*z* (feature with higher retention time)—*m*/*z* (feature with lower retention time). For each pair of features, intensity correlation across the 36 samples was also calculated. Matching observed *m*/*z* differences with mass differences in the ruleset was performed with an accepted error of ±0.002 Da. The network was visualized using Cytoscape 3.9.1 [50].

### 4.8. Time-Course Clustering

The time series were hierarchically clustered based on the shape-based distance (sbd), using the tsclust() function from the dtwclust R package version 6.0.0 [51].

## 5. Conclusions

Through the combined application of stable isotopic labeling, multistage MS^n^, Candidate Substrate–Product Pair network construction, and high temporal resolution time-course analyses, we shed light on the phenylpropanoid metabolism in flax seedlings, revealing a previously unrecognized accumulation of chicoric acid and its biosynthetic origin. Our results show that chicoric acid biosynthesis in flax likely involves direct hydroxylations of *p*-coumaroyl tartaric acid conjugates, independent of previously described chlorogenic acid-mediated pathways. The approach bypassed traditional reliance on spectral similarity or abundance correlation, enabling robust detection and structural annotation of low-abundance metabolites. Moreover, distinct biosynthetic fluxes toward lignan and early lignification intermediates were uncovered, highlighting different roles of coniferyl alcohol pools during early seedling development. These findings not only provide new insights into phenylpropanoid pathway specialization in flax but also emphasize the high nutritional value of flax sprouts due to their unexpectedly high chicoric acid content. Our integrative methodology offers a promising framework for metabolomics-driven pathway discovery in plants.

## Figures and Tables

**Table 1:** Biochemical conversions for CSPP network generation.

Nr	*m*/*z* Difference	Conversion	Short	Elution Order	# CSPP
1	2.0157	reduction	Red.neg	1	7
2	14.0157	methylation	Met	2	2
3	15.9949	oxygenation	Oxy	1	10
4	18.0106	hydration	Hydr	1	3
5	30.0106	methoxylation	Mox	2	3
6	42.0106	acetylation	Ace	2	0
7	132.0060	tartarate	Tar	1	9
8	144.0423	1,6-anhydroglucose	anhydroGlc	1	1
9	146.0579	deoxyhexose	Rha	1	0
10	156.0420	shikimate	Shi	1	0
11	162.0528	hexose	Hex	1	7
	222.0740	hexose and acetic acid adduct	Hex.AA	1	12
12	174.0530	quinate	Qui	1	0
13	192.0270	hexaric acid	GlcA	1	4
14	26.0157	beta-oxydation	Box	1	0
	28.0224	beta-oxydation.13C2	Box.13C2	1	0
15	104.0262	benzoic acid	Ben	2	0
	104.0262	benzoic acid.13C	Ben.13C	2	0
16	120.0211	hydroxybenzoic acid	Phb	2	0
	121.0245	hydroxybenzoic acid.13C1	Phb.13C	2	0
17	146.0368	coumarate	Cou	2	8
	149.0469	coumarate.13C3	Cou.13C3	2	8
18	150.0317	vanillic acid	Van	2	0
	151.0350	vanillic acid.13C	Van.13C	2	0
19	162.0317	caffeate	Caf	2	4
	165.0418	caffeate.13C3	Caf.13C3	2	7
20	176.0473	ferulate	Fer	2	0
	179.0574	ferulate.13C3	Fer.13C3	2	0
21	178.0630	condensed.guaiacyl	condGun	2	2
	181.0731	condensed.guaiacyl.13C3	condGun.13C3	2	2
22	196.0736	guaiacyl	Gun	2	2
	199.0837	guaiacyl.13C3	Gun.13C3	2	1
23	206.0579	sinapate	Sin	2	0
	209.0680	sinapate.13C3	Sin.13C3	2	0
24	208.0735	condensed.syringyl	condSun	2	0
	211.0836	condensed.syringyl.13C3	condSun.13C3	2	0
25	226.0841	syringyl	Sun	2	0
	229.0942	syringyl.13C3	Sun.13C3	2	0
sum					92

Nr, conversion number; short, shorthand naming used in edge labels of CSPP network; elution order, relative elution behavior of product and substrate in reversed-phase HPLC: 1, product elutes earlier than substrate; 2, product elutes later than substrate; # CSPP, number of candidate substrate–product pairs observed.

**Table 2:** Structurally elucidated phenylpropanoid derivatives.

Nr	RT	Compound	Adduct	Observed *m*/*z*	Formula	Δ ppm	[Isotopologue] (Cluster)	CL
**1**	2	Caffeoyl tartaric acid	[M−H]^−^	311.0402	C_13_H_11_O_9_	−0.2	[^13^C_0_] (28)	AN1
**2**	2.2	Protocatechoyl glucose	[M−H]^−^	315.0713	C_13_H_15_O_9_	−0.9	[^13^C_0_] (29); [^13^C_1_] (29)	AN2
**3**	2.8	*p*-Hydroxybenzoic acid glucoside	[M+Ac−H]^−^	359.0977	C_15_H_19_O_10_	−0.3	[^13^C_0_] (27); [^13^C_1_] (21)	AN1
**4**	3.1	Gallic acid + glucose	[M−H]^−^	331.0662	C_13_H_15_O_10_	−0.8	[^13^C_0_] (30)	PU
**5**	3.1	Coumaroyl tartaric acid	[M−H]^−^	295.0453	C_13_H_11_O_8_	−0.1	[^13^C_0_] (5); [^13^C_3_] (6)	AN1
**6**	3.5	Coumaroyl tartaric acid	[M−H]^−^	295.0454	C_13_H_11_O_8_	−0.1	[^13^C_0_] (7); [^13^C_3_] (6)	AN1
**7**	3.6	Coumaroyl glucaric acid	[M−H]^−^	355.0662	C_15_H_15_O_10_	−0.9	[^13^C_0_] (13); [^13^C_3_] (14)	AN1
**8**	6.2	Coumaric acid glucoside glucose ester	[M+Ac−H]^−^	547.1659	C_23_H_31_O_15_	−0.8	[^13^C_0_] (21); [^13^C_3_] (21)	AN2
**9**	6.2	Caffeic acid glucoside	[M−H]^−^	341.0871	C_15_H_17_O_9_	−0.5	[^13^C_0_] (12); [^13^C_3_] (6)	AN1
**10**	6.9	Coumaric acid glucoside	[M+Ac−H]^−^	385.1131	C_17_H_21_O_10_	−1	[^13^C_0_] (12); [^13^C_3_] (15)	AN2
**11**	8.5	Ferulic acid glucoside	[M+Ac−H]^−^	415.1234	C_18_H_23_O_11_	−1.5	[^13^C_0_] (12); [^13^C_3_] (11)	AN1
**12**	8.5	Dihydro-*p*-coumaroyl glucose	[M−H]^−^	327.1077	C_15_H_19_O_8_	−0.9	[^13^C_0_] (10); [^13^C_3_] (11)	AN2
**13**	8.8	Coumaroyl glucose	[M−H]^−^	325.0924	C_15_H_17_O_8_	0.1	[^13^C_0_] (8); [^13^C_3_] (9)	AN2
**14**	8.9	Coniferin	[M+Ac−H]^−^	401.1447	C_18_H_25_O_10_	−0.1	[^13^C_0_] (16); [^13^C_3_] (2)	AN1
**15**	9	Lariciresinol diglucoside	[M+Ac−H]^−^	743.2749	C_34_H_47_O_18_	−1.8	[^13^C_0_] (16); [^13^C_3_] (2); [^13^C_6_] (2)	PU
**16**	9.4	L-Chicoric acid	[M−H]^−^	473.0718	C_22_H_17_O_12_	−0.4	[^13^C_0_] (17); [^13^C_3_] (2); [^13^C_6_] (2)	ID
**17**	9.6	*p*-Coumaric acid	[M−H]^−^	163.0398	C_9_H_7_O_3_	1.8	[^13^C_0_] (3); [^13^C_3_] (4)	ID
**18**	10.3	Coumaroyl caffeoyl tartaric acid	[M−H]^−^	457.077	C_22_H_17_O_11_	−0.1	[^13^C_0_] (17); [^13^C_3_] (11); [^13^C_6_] (11)	AN2
**19**	10.5	Lariciresinol diglucoside	[M+Ac−H]^−^	743.2744	C_34_H_47_O_18_	−2.5	[^13^C_0_] (24); [^13^C_3_] (25); [^13^C_6_] (2)	PU
**20**	10.7	G(8-O-4)G-glucose	[M+Ac−H]^−^	597.2165	C_28_H_37_O_14_	−4.0	[^13^C_0_] (1); [^13^C_3_] (2); [^13^C_6_] (2)	AN1
**21**	11.4	Pinoresinol diglucoside	[M+Ac−H]^−^	741.2587	C_34_H_45_O_18_	−2.6	[^13^C_0_] (23); [^13^C_3_] (15); [^13^C_6_] (14)	AN1
**22**	11.4	Dicoumaroyl tartaric acid	[M−H]^−^	441.0816	C_22_H_17_O_10_	−1.2	[^13^C_0_] (17); [^13^C_3_] (11); [^13^C_6_] (11)	AN2
**23**	11.8	Hydroxyphenyl acetic acid	[M−H]^−^	151.0399	C_8_H_7_O_3_	2.3	[^13^C_0_] (7); [^13^C_1_] (7)	AN2
**24**	12.6	Dicoumaroyl tartaric acid	[M−H]^−^	441.0816	C_22_H_17_O_10_	−1.2	[^13^C_0_] (18); [^13^C_3_] (19); [^13^C_6_] (19)	AN2
**25**	12.6	Dehydrodiconiferyl alcohol glucoside	[M+Ac−H]^−^	579.2074	C_28_H_35_O_13_	−0.7	[^13^C_0_] (22); [^13^C_3_] (21); [^13^C_6_] (21)	AN1
**26**	12.9	Lariciresinol glucoside	[M−H]^−^	521.2015	C_26_H_33_O_11_	−1.6	[^13^C_0_] (26); [^13^C_3_] (2); [^13^C_6_] (2)	AN1
**27**	13.9	Pinoresinol glucoside	[M−H]^−^	519.1865	C_26_H_31_O_11_	−0.2	[^13^C_0_] (20); [^13^C_3_] (21); [^13^C_6_] (15)	AN1

Nr, compound number; RT, retention time expressed in minutes; observed *m*/*z*, mean of peak *m*/*z* weighted (by intensity) across scans and averaged over different samples; formula, estimated bruto formula; Δppm, difference between observed *m*/*z* and theoretical mono-isotopic *m*/*z* corresponding to the estimated bruto formula, expressed in parts per million; cluster numbers of isotopologues refer to clusters in Figure 3 and Supplementary Figure S2; CL, confidence level of compound annotation: ID, identified by spiking with reference standard; AN1, confident 2D structure annotation based on well-known gas-phase fragmentations and match with CID spectra in-house databases; AN2, probable 2D structure annotation based on well-known gas-phase fragmentations and exclusion of other candidates; PU, putative, most likely structure, no fragmentation data available. MS^n^ spectra and mass spectral interpretation are available in Supplementary Table S1 and Supplementary Methods S1.

## Data Availability

The original contributions presented in this study are included in the article/Supplementary Material. Further inquiries can be directed to the corresponding author.

## Supplementary Materials

The following supporting information can be downloaded at https://www.mdpi.com/article/10.3390/plants14152371/s1. Figure S1. MS^n^ spectra (A,B) and putative gas-phase fragmentation pathways (C,D) of chicoric acid (A, upper plots), [^13^C_3_]-chicoric acid (A, lower plots), dicoumaroyl tartaric acid (B, upper plots), and [^13^C_6_]-dicoumaroyl tartaric acid (B, lower plots). A,B. The *m*/*z* values of red-colored product ions in the lower mass spectra are equal to those of their analogs in the upper mass spectra (also red-colored) for each compound due to the absence of ^13^C label in these ions. The *m*/*z* values of blue-colored product ions in the lower mass spectra differ from those of their analogs in the upper mass spectra due to the presence of ^13^C label and result from the elimination of a non-labeled substructure (neutral loss equal in upper and lower mass spectra). The *m*/*z* values of green-colored product ions in the lower mass spectra differ from those of their analogs in the upper mass spectra due to the presence of ^13^C label and result from the elimination of a ^13^C-labeled substructure (neutral loss in lower mass spectrum differs from analogous neutral loss in upper mass spectrum). C. For each structure, the upper and lower *m*/*z* values represent the product ions in the MS^n^ spectra of chicoric acid and [^13^C_3_]-chicoric acid, respectively. D. For each structure, the upper and lower *m*/*z* values represent the product ions in the MS^n^ spectra of dicoumaroyl tartaric acid and [^13^C_6_]-dicoumaroyl tartaric acid, respectively. Figure S2. Hierarchical clustering of light and heavy isotopologue time-course profiles for 27 structurally characterized compounds. Features include light (^12^C) isotopologues and heavy (^13^C_1_, ^13^C_3_, or ^13^C_6_) isotopologues of the 27 structurally characterized compounds listed in Table 2. Time-course profiles were constructed using average intensity values across three biological replicates for each feature at each time point (t). Values are z-normalized before clustering. Time points 0 to 11 correspond to the time directly before feeding, 72 h after imbibition (time point 0), and 0.5 h, 1 h, 1.5 h, 2 h, 3 h, 4 h, 6 h, 8 h, 12 h, 24 h, and 36 h after the start of feeding with *p*-coumaric acid-1,2,3-^13^C_3_ (time points 1 to 11). Table S1. Mass spectral data of structurally elucidated phenylpropanoid derivatives. Nr, compound number; RT, retention time expressed in minutes; observed *m*/*z*, mean of peak *m*/*z* weighted (by intensity) across scans and averaged over different samples; formula, estimated bruto formula; Δppm, difference between observed *m*/*z* and theoretical mono-isotopic *m*/*z* corresponding to the estimated bruto formula, expressed in parts per million; SIL, stable isotope-labeled analogs detected; MS^n^, mass spectral fragmentation obtained at nominal mass resolution (ion trap), where relative intensities of fragment ions are indicated between brackets. Methods S1. Structural elucidation of MS^n^ spectra. Methods S2. LC–MS data processing.
