# Supplementary material for: Combining Stable Isotope Labeling and Candidate Substrate–Product Pair Networks Reveals Lignan, Oligolignol, and Chicoric Acid Biosynthesis in Flax Seedlings (Linum usitatissimum L.)
Source: Plants (Basel). 2025 Aug 1;14(15):2371. doi: 10.3390/plants14152371 (PMC12349070; doi:10.3390/plants14152371)
Supplement: Supplementary file 1 [file plants-14-02371-s001.zip › SupplementaryMehtods_S2_lcms_data_processing.pdf]

LC–MS data processing

The following functions are from the Bioconductor packages *xcms* and *CAMERA*. Unless otherwise specified, default parameters were used.

{xcms}

Peak detection on full dataset

findChromPeaks()

Specified parameter settings:

CentWaveParam(peakwidth = c(20, 80), integrate = 2)

chunkSize = 2

Chromatographic peak refinement (to reduce potential centWave-specific peak detection artifacts).

refineChromPeaks()

param = MergeNeighboringPeaksParam()

MergeNeighboringPeaksParam()

Specified parameter settings:

expandRt = 9.67525

expandMz = 0.0006

Peak grouping 1

PeakDensityParam()

sampleGroups = timepoint,

bw = 6

minFraction = 0.5

groupChromPeaks()

param = PeakDensityParam()

Retention time alignment

PeakGroupsParam()

minFraction = 0.9,

extraPeaks = 100,

smooth = "loess",

span = 0.4,

family = "gaussian"

adjustRtime()

param = PeakGroupsParam()

Peak grouping 2

PeakDensityParam()

sampleGroups = timepoint,

bw = 2

minFraction = 0.5

groupChromPeaks()

param = PeakDensityParam()

{CAMERA}

Extract peak table

filterMsLevel()

msLevel = 1L

as()

Class = xcmsSet

xsAnnotate()

Group into pseudospectra groups

groupFWHM()

perfwhm= 0.3

Annotate natural isotope peaks

findIsotopes()

maxcharge = 2

maxiso = 2

ppm = 5

minfrac = 0.5

groupCorr()

cor_eic_th = 0.75

create isotope matrix for 1, 2, 3, 4, and 5 incorporations of labeled coumaric acid (3 times 1.003355)

isoMax <- matrix(NA, 5, 4);

colnames(isoMax) <- c("mzmin", "mzmax", "intmin", "intmax")

isoMax[1,] <- c((1.003355 *3)-0.0005, (1.003355 *3)+0.0005, 1, 150)

isoMax[2,] <- c((1.003355 *6)-0.0005, (1.003355 *6)+0.0005, 1, 150)

isoMax[3,] <- c((1.003355 *9)-0.0005, (1.003355 *9)+0.0005, 1, 150)

isoMax[4,] <- c((1.003355 *12)-0.0005, (1.003355 *12)+0.0005, 1, 150)

isoMax[5,] <- c((1.003355 *15)-0.0005, (1.003355 *15)+0.0005, 1, 150)

Annotate SIL isotope peaks

findIsotopes()

mzabs = 0.005

ppm = 5

maxiso = 4

filter = FALSE

maxcharge = 1

minfrac = 0.01

isotopeMatrix = as.matrix(isoMax)
