# Supplementary material for: Combining Stable Isotope Labeling and Candidate Substrate–Product Pair Networks Reveals Lignan, Oligolignol, and Chicoric Acid Biosynthesis in Flax Seedlings (Linum usitatissimum L.)
Source: Plants (Basel). 2025 Aug 1;14(15):2371. doi: 10.3390/plants14152371 (PMC12349070; doi:10.3390/plants14152371)
Supplement: Supplementary file 1 [file plants-14-02371-s001.zip › SupplementaryMethodsS1_revision26072025.pdf]

Structural elucidation of MSn spectra.

1

LC–MS features: M311.04T561.66

The ion at m/z 311 eluting at 2.0 min had the molecular formula C13H11O9. No [13C3]-labeled analog was found for this compound. The MS2 spectrum of this ion was characterized by two main fragment ions at m/z 179 and 149. Further MS3 fragmentation of the first product ion at m/z 179 resulted in the loss of a carboxylic acid moiety (neutral loss of 44 Da) and, to a lesser extent, the loss of a water molecule (neutral loss of 18 Da). If we identify this first product ion at m/z 179 as the carboxylate anion of caffeic acid, the eliminated substructure corresponds to the molecular formula [C13H12O9 – C9H8O4 + H2O = C4H6O6]. The MS3 spectrum of the first product ion at m/z 149, characterized by fragments at m/z 59, 73, 87, 103 (base), 105, and 131, was searched for in an in-house spectral library. A perfect match was obtained with tartaric acid (C4H6O6) (authentic standard), yielding a hit (number of common fragments) and score (dot product) of 6 and 0.9, respectively. In the MS2 spectrum, we, therefore, annotated the fragments at m/z 179, 131 (trace), and 149 as the caffeic acid carboxylate anion, anhydrotartaric acid, and the tartaric acid alkoxide anion, respectively, typical for the two cleavage types of hydroxycinnamoyl esters. The ynolate anion of caffeic acid was not detected, however. We annotated this compound as **caffeoyl tartaric acid**.

2

LC–MS features: M315.071T133.17, M316.075T132.85

The ion at m/z 315 eluting at 2.2 min had the molecular formula C13H15O9. The MS2 base peak at m/z 153 resulted from anhydrohexose loss (-162 Da), and the MS3 spectrum showed that decarboxylation, yielding the ion at m/z 109, was the main fragmentation pathway for the m/z 153 ion. MS2 ions at m/z 225, 195, and 165, resulting from hexose cross-ring cleavages, indicated that the hexose was connected as a hexoside with a free reducing end or via an ester bond (see *p*-coumaric acid). The aglycone is a dihydroxybenzoic acid. In young flax seedlings, 3,4-dihydroxybenzoic acid (protocatechuic acid) has been reported (Huang et al., 2021). Moreover, in the case of a 2,4-dihydroxybenzoic acid moiety (homogentisic acid), the MS3 spectrum of this moiety should show a base peak due to water loss, which is not observed here (Morreel et al., 2014). The MS3 spectrum of the first-order ion at m/z 153 matched with protocatechuic acid (authentic standard) in our in-house spectral library. Therefore, **protocatechoyl glucose** was proposed as the structure for this molecule. Further support for the ester bond was provided by searching the complementary pairs of ions associated with the two characteristic cleavages that esters undergo (Morreel et al., 2014, Debrauwer et al., 1992, Fournier et al., 1993, Fournier et al., 1995, Stroobant et al., 1995). The first charge-remote cleavage type produces a carboxylate anion and a neutral, which remain initially together in an ion–dipole complex. The complex can then dissociate or might be preceded by a proton transfer between the carboxylate anion and the neutral, hence leading to the loss of a neutral carboxylic acid. This complementary pair of ions was observed at m/z 153 (carboxylate ion, base peak) and 161 (anhydrohexose ion, trace). A second cleavage type, which occurs to a lesser extent, produces a neutral ketene and an alkoxide anion that remain together in an ion–dipole complex. Again, the complex can dissociate as such or might be preceded by a proton transfer yielding an ynolate ion and the neutral alcohol. This complementary pair of ions was observed at m/z 135 (ynolate ion) and 179 (alkoxide ion).

3

<u>LC–MS features:</u> M299.076T168.68, M359.098T168.86, M360.101T169

The ion at m/z 299 eluting at 2.8 min had the molecular formula C13H15O8. This compound was more readily ionized as an acetate adduct at m/z 359 (C15H19O10). CID fragmentation of the adduct at m/z 359 led to the loss of acetate, leading to the ion at m/z 299, and MS3 fragmentation of this first product ion led to the loss of anhydrohexose, resulting in the fragment at m/z 137 and a minor fragment at m/z 93 due to additional loss of carboxylic acid (44 Da). The fragmentation pattern and molecular formula correspond to ***p*-hydroxybenzoic acid glucoside** in our in-house spectral library.

4

<u>LC–MS features:</u> M331.066T184.04

The ion at m/z 331 eluting at 3.1 min had the molecular formula C13H15O10. No fragmentation was obtained for this compound. The molecular formula corresponds to a gallic acid aglycone connected to a hexose. Because gallic acid has been described in young flax seedlings, we proposed **gallic acid glucoside or galloyl glucose** for this compound (Huang et al., 2021).

5, 6

<u>LC–MS features:</u> M295.045T210.44, M298.055T211.03, M295.045T186.07, M298.055T186.08

The ion at m/z 295 eluting at 3.1 min had the molecular formula C13H11O8. The m/z value of the base peak in the MS2 spectrum of the unlabeled compound (m/z 163) differed from the one in the MS2 spectrum of the analogous labeled compound (m/z 166) owing to the presence of a [13C3]-labeled moiety. This main product ion thus results from the elimination of a non-labeled substructure. MS3 fragmentation of these ions (m/z 163 and m/z 166) resulted in the neutral loss of 44 Da and 45 Da, respectively, owing to the $^{12}CO_2$ or $^{13}CO_2$ loss of the non-labeled or [13C3]-labeled precursor ion. If we identify the fragment at m/z 163 or m/z 166 in the MS2 spectrum as unlabeled or [13C3]-labeled ***p*-coumaric acid** (C9H8O3), the eliminated substructure corresponds to the molecular formula [C13H12O8 - C9H8O3 + H2O = C4H6O6], which corresponds to **tartaric acid**. The m/z values of fragment ions at m/z 149, 131, 113, and 87 were common in the MS2 spectrum of the unlabeled compound and its labeled analog and therefore resulted from the elimination of a [13C3]-labeled substructure. The fragment at m/z 149 corresponded to the molecular ion of tartaric acid and the fragments at m/z 87, m/z 103, and m/z 131 are typical for the fragmentation spectra of tartaric acid obtained by Q-TOF or QQ mass spectrometry reported in MassBank and in ion trap-generated spectra of an authentic standard in our in-house database. The common fragment ion at m/z 113 corresponds to anhydrotartaric acid and can be attributed to the typical cleavage of hydroxycinnamoyl esters: The most prevalent cleavage produces a carboxylate anion and a neutral, which remain initially together in an ion–dipole complex. The complex can then dissociate or might be preceded by a proton transfer between the carboxylate anion and the neutral, hence leading to the loss of a neutral carboxylic acid. This complementary pair of ions was observed at m/z 163 or m/z 166 (*p*-coumaric acid carboxylate ion, base peak) and 131 (anhydrotartaric acid ion, less abundant).

An isomer of this compound showing the same fragmentation pattern also eluted at 3.5 min.

These compounds were annotated as two distinct isomers of ***p*-coumaroyl tartaric acid**.

7

LC–MS features: M355.066T215.91, M358.076T214.84

The ion at m/z 355 eluting at 3.6 min had the molecular formula C15H15O10. Both the unlabeled ion and its [13C3]-labeled counterpart at m/z 358 showed the loss of a water molecule (-18 Da) in the MS2 spectrum and had a common MS2 base peak at m/z 191 and an additional common strong MS2 fragment at m/z 209. These two common product ions thus resulted from the elimination of a [13C3]-labeled substructure, which we annotated as *p*-coumaric acid (neutral losses of 164 Da in the unlabeled and 176 Da in the labeled compound) and dehydrated *p*-coumaric acid (neutral losses of 146 Da in the unlabeled and 149 Da in the labeled compound). The MS2 spectrum, as well as the MS3 fragmentation, of the first product ions at m/z 191 and m/z 209 were exactly as previously analyzed in our lab for *p*-coumaroyl glucaric acid in *Isatis tinctoria* (Nguyen et al., 2017). In *Isatis tinctoria*, the identity of the glucaric acid moiety had been confirmed by feeding with 13C6-labeled glucaric acid. Therefore, we annotated this compound as ***p*-coumaroyl glucaric acid**.

8

LC–MS features: M547.166T372.5, M550.175T369.34

The ion at m/z 547 eluting at 6.2 min had the molecular formula C23H31O15. A [13C3]-labeled analog was also detected; this compound was therefore derived from *p*-coumaric acid containing all three aliphatic carbon atoms. The MS2 spectrum showed intense peaks at m/z 487, resulting from the loss of an acetate adduct, at m/z 325 due to the combined loss of acetate and anhydrohexose, and a minor fragment at m/z 163 due to the combined loss of acetate and two anhydrohexoses. These neutral losses were identical in the MS2 spectrum of the [13C3]-labeled analog, confirming that the expelled moieties were not 13C-labeled. The compound is an acetate adduct of an aglycone attached to two hexoses, and the aglycone has the molecular formula C9H8O3 (C23H31O15 +H – C2H4O2 – C6H10O5 – C6H10O5 = C9H8O3), which corresponds to *p*-coumaric acid. Because the loss of a single anhydrohexose (m/z 325) was much more prominent than the combined loss of two anhydrohexoses, the aglycone (*p*-coumaric acid) probably carried two hexose moieties at distinct positions, rather than one dihexose. The MS2 spectrum of the [13C3]-labeled analog also showed the carbohydrate cross-ring cleavages typical for ester-linked glucoses (losses of 60, 90, and 120 Da from the base peak at m/z 490: m/z 430, m/z 400, and m/z 370). Therefore, we annotated this compound as ***p*-coumaric acid glucoside glucose ester**.

9

LC–MS features: M341.087T373.13, M344.097T373.55

The ion at m/z 341 eluting at 6.2 min had the molecular formula C15H17O9. The MS2 spectra of both this ion and its [13C3]-labeled analog were characterized by the loss of an anhydrohexose moiety (162 Da) (fragment at m/z 179 and m/z 182 in the unlabeled and [13C3]-labeled compound, respectively). An ynolate ion was not observed, and only the traces of hexose cross-ring cleavages (losses of 120, 90, and 60 Da) could be distinguished in certain MS2 spectra of this ion. Therefore, this compound is probably not a hydroxycinnamoyl ester but rather a hexoside. The MS3 spectrum of the first product ion at m/z 179 led to a fragment at m/z 135 due to carboxylic acid loss (44 Da). This latter fragment was shifted to m/z 137 in the MS3 spectrum of the first product ion at m/z 182 of the [13C3]-labeled analog. This is in accordance with the loss of a [13C1]-labeled carboxylic acid moiety (45 Da) from a [13C3]-labeled hydroxycinnanmic acid. This MS3 spectrum corresponds to the fragmentation spectrum of an authentic caffeic acid standard in our in-house database. This compound was annotated as **caffeic acid glucoside**.

10

LC–MS features: M385.113T415.18, M388.123T415.33, M651.191T416.82, M654.201T416.06, M657.212T416.06

The ion at m/z 385 eluting at 6.9 min had the molecular formula C17H21O10. The MS2 spectra of both this ion and its [13C3]-labeled analog were characterized by the loss of 60 Da (fragment at m/z 325 and m/z 328 in the unlabeled and [13C3]-labeled compound, respectively) and the combined losses of 60 Da and 162 Da (fragment at m/z 163 and m/z 166 in the unlabeled and [13C3]-labeled compound, respectively), indicating that this was an acetic acid adduct of a hexoside. The MS3 spectrum of the first product ion at m/z 325 led to a major fragment at m/z 163 due to anhydrohexose loss and a minor fragment at m/z 119 due to additional carboxylic acid loss. This latter fragment was shifted to m/z 121 in the MS3 spectrum of the first product ion at m/z 328 of the [13C3]-labeled analog. This compound was annotated as ***p*-coumaric acid glucoside**.

The ion at m/z 651 eluting at 6.9 min had the molecular formula C30H35O16. A [13C3]-labeled analog and a [13C6]-labeled analog were also strongly represented, indicating that this compound was derived from two *p*-coumaric acid moieties and contained all six aliphatic carbons. The MS2 spectrum of the unlabeled compound was dominated by a fragment at m/z 325. This fragment contained three aliphatic *p*-coumaric acid carbons, since, in the MS2 spectrum of the [13C3]-labeled analog, it was represented by a doublet at m/z 325 – m/z 328, and in the MS2 spectrum of the [13C6]-labeled analog, this fragment was at m/z 328. Further MS3 fragmentation of this first product ion invariably led to the loss of 162 Da, corresponding to anhydrohexose, and therefore, the fragment at m/z 325 or m/z 328 was annotated as *p*-coumaric acid glucoside. The second most intense peak in the MS2 spectrum of this compound was at m/z 489, m/z 492, and m/z 495 for the unlabeled, [13C3]-labeled, and [13C6]-labeled compounds, respectively, and was also due to the invariable neutral loss of an unlabeled 162 Da. Finally, a fragment at m/z 487 in the MS2 spectrum of the unlabeled compound was shifted to m/z 490 in the MS2 spectrum of the [13C6]-labeled analog and corresponded to the loss from the molecular ion of unlabeled or labeled *p*-coumaric acid, respectively. This compound had the same retention time as feature M385.113T415.18, which was annotated as an acetic acid adduct or *p*-coumaric acid glucoside, and the [13C3]- and [13C6]-labeled analogs of this compound showed the same accumulation profile as the [13C3]-labeled analog of the *p*-coumaric acid glucoside acetate adduct (M388.123T415.33). Therefore, we identify these features as an adduct of two ***p*-coumaric acid glucoside** compounds.

11

LC–MS features: M415.123T511.52, M418.134T511.52, M714.222T510.96, M717.232T510.73

The ion at m/z 415 eluting at 8.5 min had the molecular formula C18H23O11 and was also detected as a homodimer $[2M-H]^-$ at m/z 711. The [13C3]-labeled analog was detected as an ion at m/z 418 and, more intensely, as an adduct of the unlabeled and [13C3]-labeled analogs at m/z 714 or as an adduct of two [13C3]-labeled compounds at m/z 717. The MS2 spectrum of the [13C3]-labeled compound at m/z 418 showed a base peak at m/z 358, indicating that the ions at m/z 415 and m/z 418 were acetate adducts of compounds of mass 356 Da (unlabeled) and 359 Da ([13C3]-labeled), respectively. The MS2 fragmentation of the heterodimer at m/z 714 resulted, indeed, in two base peaks at m/z 355 and m/z 358. The MS3 spectra of these first product ions at m/z 355 and m/z 359 both showed a loss of an anhexoside (neutral loss of 162 Da), resulting in base peaks at m/z 193 and m/z 196, respectively. The MS4 spectrum of the second product ion at m/z 196, which corresponded to a [13C3]-labeled fragment, was characteristic for ferulic acid (in-house spectral database), with neutral losses of 15 Da (fragment at m/z 181), indicative of a methoxyl group on the phenyl ring, and of 45 Da (m/z 151) corresponding to a [13C1]-labeled carboxylic acid moiety, and the combination of these two losses (m/z 136). This compound was therefore annotated as **ferulic acid glucoside**.

12

LC–MS features: M327.108T511.82, M330.118T510.98, M686.228T512.17, M689.237T511.03

The ion at m/z 327 eluting at 8.5 min had the molecular formula C15H19O8, and a [13C3]-labeled analog was detected at m/z 330. This compound was ionized more intensely under the form of an adduct of a homo-dimer with CO. The homodimer CO adduct contained two unlabeled forms (C15H19O8 + C15H20O8 + CO = C31H39O17) (ion at m/z 683 and MS2 spectrum with base peak at m/z 327), an unlabeled and a [13C3]-labeled analog (ion at m/z 686 and MS2 spectrum with equally intense peaks at m/z 327 and m/z 330), or two [13C3]-labeled analogs (ion at m/z 689 and MS2 spectrum with base peak at m/z 330). MS3 fragmentation of the ion at m/z 327 or the [13C3]-labeled ion at m/z 330 invariably led to the loss of an anhyrdohexose moiety (neutral loss of 162 Da), leading to a fragment at m/z 165 or m/z 168 in the case of the labeled analog. MS4 fragmentation of the second product ion at m/z 165 led to the neutral loss of 44 Da, corresponding to the loss of a carboxylic acid moiety. The loss of this carboxylic acid moiety from the [13C3]-labeled second product ion at m/z 168 corresponded to a neutral loss of 45 Da, indicating this moiety was the carboxylic acid end group of a derivative of *p*-coumaric acid. We annotated this compound as **dihydro-*p*-coumaroyl glucose**.

13

LC–MS features: M325.092T530.52, M328.103T528.02

The ion at m/z 325 eluting at 8.8 min had the molecular formula C15H17O8. The [13C3]-labeled analog of this compound was more abundant than the unlabeled form in all samples, and MSn fragmentation was

only obtained from the [13C3]-labeled analog at m/z 328. Elimination of an anhydrohexose moiety led to the base peak at m/z 166. First product ions at m/z 208, 238, and 268 due to hexose cross-ring cleavages (losses of 120, 90, and 60 Da) indicated that the hexose was linked in an ester bond (see Dauwe et al. (2007) for feruloyl hexose and sinapoyl hexose and Vanholme et al. (2010) for 5-hydroxyferuloyl hexose (Dauwe et al., 2007, Vanholme et al., 2010)) or that it was a hexoside with a free reducing end (Carroll et al., 1995, Mulroney et al., 1999, March and Stadey, 2005). An ester bond was confirmed by its characteristic second type of cleavage, in which an ynolate ion was formed at m/z 148. Indeed, a hydroxycinnamoyl ester ion often generates an ynolate ion upon CID (Debrauwer et al., 1992, Fournier et al., 1995, Fournier et al., 1993). This compound was annotated as ***p*-coumaroylglucose**.

14

LC–MS features: M401.145T536.79, M404.154T535.23

The ion at m/z 401 eluting at 8.9 min had the molecular formula C18H25O10. A [13C3]-labeled analog was also detected; this compound was therefore derived from *p*-coumaric acid, containing all three aliphatic carbon atoms. Its MS2 spectrum showed a peak at m/z 341, resulting in the loss of acetate (-60 Da; $[M-H-C2H4O2]^-$) and was dominated by the combined loss of the acetate anhydrohexose, yielding the first product ion at m/z 179. The feature at m/z 401 was, thus, an acetate adduct of a hexoside of which the aglycone had the molecular formula C18H25O10 +H – C2H4O2 – C6H10O5 = C10H12O3 (m/z 179). Further MS3 fragmentation of this first product ion at m/z 179 led to second product ions at m/z 161 (base peak, water loss), 164 (loss of methyl radical), and 146 (combined loss of water and methyl radical). The MS4 spectrum of the second product ion at m/z 161 was dominated by the loss of a methyl radical, resulting in a fragment at m/z 146. The fragmentation is identical to the fragmentation of coniferin acetate adduct described in Arabidopsis vacuoles (authenticated spectra obtained previously in our lab and stored in our in-house database) (Dima et al., 2015). This compound was annotated as **coniferin**.

15, 19

LC–MS features: M743.275T537.5, M746.285T536.79, M749.295T511.23, M743.274T628.48, M746.289T626.91, M749.295T600.21

The ion at m/z 743 eluting at 8.9 min had the molecular formula C34H47O18, and [13C3]-labeled and [13C6]-labeled analogs of this compound were also detected, indicating the presence of two *p*-coumaric acid-derived moieties, in which all aliphatic carbon atoms were present. This ion and its two labeled analogs correspond to the acetate adduct of a triplet of ions eluting at the same time at m/z 683, 686, and 689, which had the molecular formula C32H43O16. No fragmentation was obtained for this compound or for the acetate adduct; however, based on the molecular formula and the [13C3]- and [13C6]-labeling, we tentatively annotated this compound as **lariciresinol diglucoside.**

Another acetate adduct with the same molecular formula and [13C3]- and [13C6]-labeling and for which the intensity was also too low to obtain fragmentation eluted at 10.4 min and was, likewise, tentatively annotated as **lariciresinol diglucoside**.

16

<u>LC–MS features:</u> M473.072T561.16, M476.082T561.57, M479.092T560.21

The ion at m/z 473 eluting at 9.4 min had the molecular formula C22H17O12. Both [13C3]-labeled and [13C6]-labeled analogs of this ion were also detected. For this compound, MSn spectra and putative gas-phase fragmentation pathways are illustrated in Supplementary Figure 1. The m/z value of the two most intense peaks in the MS2 spectrum of the unlabeled compound (m/z 293 and 311) differed from those in the MS2 spectrum of the [13C3]-labeled compound (m/z 296 and 314), owing to the presence of a [13C3]-labeled moiety. Further MS3 and MS4 fragmentation indicated that the first product ion at m/z 311, or m/z 314 in the [13C3]-labeled analog, corresponded to unlabeled and [13C3]-labeled caffeoyl tartaric acid, respectively, as it was highly similar to the fragmentation of free caffeoyl tartaric acid eluting at 2.0 min (see above). The MS2 fragment at m/z 293, or m/z 296 in the [13C3]-labeled analog, corresponds to the dehydrated form of caffeoyl tartaric acid. Further putative fragmentation pathways giving rise to the fragments observed in the MS3 spectrum of the first product ion at m/z 293 are given in Supplementary Figure 1. These two first product ions (caffeoyl tartaric acid and anhydrocaffeoyl tartaric acid) can arise from the two alternative cleavage mechanisms of a hydroxycinnamoyl ester of caffeoyl tartaric acid. The identity of the ester-linked moiety was provided by searching the complementary pairs of ions associated with the two characteristic hydroxycinnamoyl ester cleavages. A first complementary pair of ions was observed at m/z 179 (caffeic acid carboxylate ion) and 293 (anhydrocaffeoyl tartaric acid). The second complementary pair of ions was observed at m/z 161 (caffeic acid ynolate ion) and 311 (caffeoyl tartaric acid alkoxide ion). For all fragments containing a caffeic acid moiety (m/z 161, 179, 219, 293, 311; see Supplementary Figure 1), the phenylpropanoid structure was confirmed by the appearance of an additional [13C3]-labeled form (with m/z value shifted 3 Da upward) in the MS2 spectrum of the [13C3]-labeled compound. The caffeic acid carboxylate ion further lost a carboxylic acid moiety, resulting in a fragment at m/z 135 in the MS2 spectrum of the unlabeled compound, and this structure was confirmed by the appearance of a [13C2]-labeled product ion at m/z 137 in the MS2 spectrum of the [13C3]-labeled analog. The m/z value of a first product ion at m/z 149 in the MS2 spectrum of the unlabeled compound was unaltered in the MS2 spectrum of the [13C3]-labeled analog, and MS3 fragmentation confirmed that this fragment corresponded to tartaric acid. We identified this compound as dicaffeoyl tartaric acid, which is also called **chicoric acid**. Spiking with an authentic standard showed that this compound was the L-isomer: **L-chicoric acid**.

17

<u>LC–MS features:</u> M163.04T576.32, M166.05T576.06

The ion at m/z 163 eluting at 9.6 min had the molecular formula C9H7O3. The MS2 spectrum was dominated by a fragment at m/z 119 due to the elimination of a carboxylic acid group. In the MS2 spectrum of the [13C3]-labeled analog of this compound, this product ion at m/z 119 was shifted to m/z 121, corresponding to the loss of a [13C1]-labeled carboxylic acid moiety. We confirmed the identification of this compound as ***p*-coumaric acid** by spiking with the authentic standard.

18

LC–MS features: M457.077T620.2, M460.087T619.84, M463.097T620.2

The ion at m/z 457 eluting at 10.3 min had the molecular formula C22H17O11. Both the [13C3]-labeled analog and the [13C6]-labeled analog were also observed but remained at trace levels, and MSn fragmentation was only obtained for the unlabeled compound at m/z 457. The MS2 spectrum of this ion shows fragments that correspond to para-coumaric acid and caffeic acid carboxylate ions (m/z 163 and m/z 179), para-coumaroyl tartaric acid and caffeoyl tartaric acid (m/z 295 and m/z 311), coumaroyl anhydrotartaric acid and caffeoyl anhydrotartaric acid (m/z 277 and m/z 293), and fragments of these latter compounds (m/z 203 and m/z 219). Further fragmentation of this compound was analogous to the fragmentation observed for dicaffeoyl tartaric acid **(16)** and for dicoumaroyl tartaric acid (**22**, **24**). We annotated this compound as ***p*-coumaroyl-caffeoyl tartaric acid**.

20

LC–MS features: M597.216T639.67, M600.229T640, M603.237T640

The ion at m/z 597, with molecular formula C28H37O14, eluted under the form of twin peaks with similar intensities at 10.5 and 10.10 min. Of these two compounds, a [13C3]-labeled (m/z 600) and a [13C6]-labeled analog (m/z 600 and m/z 603) were also detected. These compounds were therefore derived from two molecules of *p*-coumaric acid, containing all six aliphatic carbon atoms. The peak-picking algorithm did not resolve the two twin peaks so that they are both represented by features M597.216T639.67, M600.229T640, and M603.237T640.

For both compounds, the MS2 spectrum showed peaks at m/z 536, resulting from the loss of an acetate adduct, and at m/z 375 due to the combined loss of acetate and anhydrohexose. These neutral losses were identical in the MS2 spectrum of the [13C3]- and [13C6]-labeled analogs, confirming that the expelled moieties were not 13C-labeled. The compounds are acetate adducts of a hexoside, of which the aglycone has molecular formula C20H24O7 (C28H37O14 +H – C2H4O2 – C6H10O5 = C20H8O3) and contains two *p*-coumaric acid-derived moieties. The aglycone ion (m/z 375, m/z 378, and m/z 380 in the unlabeled, [13C3]-, and [13C6]-labeled analogs, respectively) was further fragmented to ions at m/z 327, 195, and 179 in the MS3 spectrum. These are due to a combined water/formaldehyde loss (- 48 Da, m/z 327), characteristic of the 8–O–4-linkage in (neo)lignans/oligolignols, and cleavage of this linkage, resulting in second product ions representing each of the units in this dimer (Morreel et al., 2010). In the fragmentation of the aglycone of the second isomer (eluting at 10.10 min), the individual neutral losses of a water molecule (18 Da) and of formaldehyde (-30 Da) resulted in major fragment ions, whereas the combined neutral loss of water and formaldehyde (- 48 Da) was relatively less intense. These fragmentation patterns have been well described before and are characteristic for the *threo* and the *erythro* forms of the 8–O–4-linkage in (neo)lignans/oligolignols, for which the reference spectra of authenticated standard compounds are available in our in-house spectral database (Morreel et al., 2010). The compounds eluting at 10.5 and 10.10 are annotated, based on MSn elucidation and MS3 spectral identity, as the *threo* isomer and the *erythro* isomer of guaiacylglycerol 8–O–4 coniferyl ether hexoside, respectively, shortly written as **G(*t*8–O–4)G hex** and **G(*e*8–O–4)G hex**.

21

LC–MS features: M741.259T683.43, M744.269T681.6, M747.28T681.6

The ion at m/z 741 eluting at 11.4 min had the molecular formula C34H45O18. A [13C3]-labeled analog and a [13C6]-labeled analog were also observed, indicating that this compound was derived from two *p*-coumaric acid skeletons. Its MS2 spectrum showed the loss of an acetate adduct (-60 Da; $[M-H-C2H4O2]^-$), rendering the peak at m/z 681, which has therefore the molecular formula C34H45O18 - C2H4O2 = C32H41O16. In the MS1 spectrum, an ion at m/z 681, with molecular formula C32H41O16, was indeed also observed, as well as the [13C3]- and [13C6]-labeled analogs hereof. Upon CID of the ion at m/z 681, a hexose loss was observed, leading to the ion at m/z 519. MS3 fragmentation of this first product ion rendered the peak at m/z 357 due to a second hexose loss. The MS4 spectrum of this second product ion was identical to the MS2 spectrum of pinoresinol or G(8–8)G, present in our in-house spectral database (Ye et al., 2005, Guo et al., 2007, Eklund et al., 2008b, Ricci et al., 2008, Morreel et al., 2010, Hanhineva et al., 2012). Therefore, this compound is **pinoresinol dihexoside**. No CID fragmentation of the [13C3]- or [13C6]-labeled analogs was obtained.

22, 24

LC–MS features: M441.082T686.75, M444.092T687.91, M447.102T686.78, M441.082T753.42, M444.092T755.68, M447.102T757.87

The ion at m/z 441 eluting at 12.6 min had the molecular formula C22H17O10. Both the [13C3]-labeled analog and the [13C6]-labeled analog were also observed, and MSn fragmentation was obtained for the unlabeled compound and for the [13C6]-labeled analog at m/z 447. MSn spectra and putative gas-phase fragmentation pathways are illustrated in Supplementary Figure 1. The m/z value of the two most intense peaks in the MS2 spectrum of the unlabeled compound (m/z 277 and 295) differed from those in the MS2 spectrum of the [13C6]-labeled compound (m/z 280 and 298), owing to the presence of a [13C3]-labeled moiety in these fragments. MS3 fragmentation of both the first product ion at m/z 295 of the unlabeled compound and the [13C3]-labeled first product ion at m/z 298 of the [13C6]-labeled analog resulted in common fragments at m/z 149, 131, and 113, which were therefore not derived from a phenylpropanoid side chain and corresponded to tartaric acid, anhydrotartaric acid, and di-anhydrotartaric acid. The base peak of the MS3 spectrum of the first product ion at m/z 295 of the unlabeled compound was at m/z 163 and shifted to m/z 166 in the MS3 spectrum of the [13C3]-labeled first product ion at m/z 298. This fragment corresponds to unlabeled or [13C3]-labeled *p*-coumaric acid. A fragment at m/z 119 in the MS3 spectrum of the first product ion at m/z 295 of the unlabeled compound was shifted two Da upward to m/z 121 in the MS3 spectrum of the [13C3]-labeled first product ion at m/z 298 of the labeled analog and corresponded to decarboxylated *p*-coumaric acid.

The MS2 fragment at m/z 277, or m/z 280 for the [13C6]-labeled analog, corresponds to the dehydrated form of *p*-coumaroyl tartaric acid. Further putative fragmentation pathways giving rise to the fragments (m/z 113, 131, 145, 203 (base), and 259) observed in the MS3 spectrum of the first product ion at m/z 277, and to the fragments at m/z 174 and m/z 175 in the MS4 spectrum of the second product ion at m/z 203, are given in Supplementary Figure 1.

These two first product ions (*p*-coumaroyl tartaric acid and anhydro-*p*-coumaroyl tartaric acid) can arise from the two alternative cleavage mechanisms of a hydroxycinnamoyl ester of *p*-coumaroyl tartaric acid. The identity of the ester-linked moiety was provided by searching the complementary pairs of ions

associated with the two characteristic hydroxycinnamoyl ester cleavages. A first complementary pair of ions was observed at m/z 163 (para-coumaric acid carboxylate ion) and 277 (anhydro-para-coumaroyl tartaric acid). The second complementary pair of ions was observed at m/z 145 (para-coumaric acid ynolate ion) and 295 (para-coumaroyl tartaric acid alkoxide ion).

Another isomer of this compound, with [13C3]- and [13C6]-labeled analogs showing a highly similar accumulation profile over the studied time course and with a highly similar MSn fragmentation, eluted at 11.4 min. These compounds eluting at 12.6 and 11.4 min were annotated as two distinct isomers of **di-*p*-coumaroyl tartaric acid**.

23

LC–MS features: M151.04T709.72, M152.043T709.93

The ion at m/z 151 eluting at 11.8 min had the molecular formula C8H7O3. This molecular formula corresponds to vanillin, which has been previously reported in germinating flaxseeds (Huang et al., 2021). However, the main CID fragmentation pathway of this compound led to the loss of 44 Da, indicating a carboxylic acid (whereas vanillin is an aldehyde), and no methyl radical loss was observed, as could be expected for vanillin. Therefore, we annotated this compound as **hydroxyphenylacetic acid**.

25

LC–MS features: M579.207T757.61, M582.217T743.3, M585.227T757.1, M580.211T757.61, M583.221T758.09, M586.231T757.85

The ion at m/z 579 eluting at 12.6 min had the molecular formula C28H35O13. MS2 fragmentation yielded the deprotonated compound at m/z 519, after loss of acetate (60 Da) from the acetate adduct. Other second product ions at m/z 357 (due to hexose loss) and at m/z 339 and 327 (losses of water and formaldehyde in addition to hexose)—fragmentations typically observed in the spectrum of phenylcoumarans—pointed to a hexoside of a phenylcoumaran (Morreel et al., 2010). In the MS2 spectrum of the [13C6]-labeled analog, the fragments at m/z 357 and 339 were shifted 6 Da upwards to m/z 363 and 345, confirming that the complete aliphatic side chains of two *p*-coumaric acid-derived phenylpropanoids were still present in these fragments. The first product ion at m/z 327, however, was only shifted 5 Da upwards in the fragmentation of the [13C6]-labeled analog, confirming that this fragment resulted from the loss of formaldehyde from the aliphatic side chain of one of the phenylpropanoid constituents, eliminating as such one 13C from the labeled analog. MS3 and MS4 spectra obtained from the first product ions at m/z 339 and 327 showed that, for both of these two fragments, the main gas-phase fragmentation pathways led to the successive losses of 15 Da methyl moieties, indicative of the presence of two methoxyl groups attached to the phenolic rings. In the MS2 spectrum, the type II ion at m/z 221 indicated the presence of a guaiacyl unit (Morreel et al., 2010). These spectra are in accordance with the fragmentation spectrum of an authentic dehydrodiconiferyl alcohol in our in-house spectral database. This compound was thus annotated as **dehydrodiconiferyl alcohol hexoside**.

26

<u>LC–MS features:</u> M521.201T772.74, M524.212T749.27, M527.222T773.16

The ion at m/z 521 eluting at 12.8 min had the molecular formula $C_{26}H_{33}O_{11}$. The presence of [13C3]-labeled and [13C6]-labeled analogs of this compound indicated the presence of two *p*-coumaric acid-derived moieties, in which all aliphatic carbon atoms were present. A common neutral loss of 162 Da in the MS2 spectrum of both unlabeled and [13C3]-labeled or [13C6]-labeled analogs pointed to a hexoside. Further MS3 fragmentation led to a neutral loss of 30 Da from the unlabeled aglycone (primary product ion at m/z 329) but a neutral loss of 31 Da from the [13C6]-labeled aglycone (primary product ion m/z 334), and this fragmentation was already observed in the MS2 spectra of both the unlabeled and [13C6]-labeled compounds, where m/z 329 and m/z 334 were, respectively, the base peaks. It is typical for lignans containing hydroxymethyl groups, and particularly for lariciresinol, to very easily lose formaldehyde in the negative ion mode (Eklund et al., 2008a, Thiombiano et al., 2020). The difference in neutral loss between the unlabeled and [13C6]-labeled analog (31 Da *versus* 30 Da) confirms that the eliminated group contains one carbon atom originating from the aliphatic side chain of one of the phenylpropanoid constituents. Further MS3 fragmentation of the first product ion at m/z 329 and MS4 fragmentation of its most intense second product ion at m/z 178 corresponded to the fragmentation earlier described for lariciresinol (Eklund et al., 2008a) and the fragmentation of a lariciresinol standard in our in-house spectral database. We, therefore, annotated this compound as **lariciresinol monoglucoside**. The shifts in m/z values observed between the MSn fragments of the unlabeled compound and those observed in the MSn fragmentation of the [13C6]-labeled analog indicated the number of aliphatic phenylpropanoid carbon atoms in the fragments, which allowed us to assign structures to these fragments and fragmentation pathways with a high confidence level. Mutual mass differences of 15 Da between pairs of product ions in the MS3 spectrum of the first product ion at m/z 329 result from the expelling of a methyl radical due to homolytic cleavage and can be considered as a spectral fingerprint indicative of the presence of methoxylated phenyl rings.

27

<u>LC–MS features:</u> M519.187T835.82, M522.196T835.82, M525.207T835.18

The ion at m/z 519 eluting at 13.9 min had the molecular formula $C_{26}H_{31}O_{11}$ and was also detected under the form of a [13C3]-labeled analog and a [13C6]-labeled analog. The MS2 spectra of this compound and of its [13C3]-labeled and [13C6]-labeled analogs all showed a primary product ion due to the elimination of the same unlabeled, neutral loss of 162 Da. This compound was therefore a hexoside of an aglycone at m/z 357 and molecular formula $C_{26}H_{31}O_{11}$ +H – $C_6H_{10}O_5$ = $C_{20}H_{22}O_6$. The MS3 spectrum of this aglycone at m/z 357 corresponded to the CID fragmentation of pinoresinol, of which the fragments have been annotated earlier, and for which reference spectra are available in our in-house spectral database (Morreel et al., 2010). The compound was thus annotated as **pinoresinol monoglucoside**. Here, the shifts in m/z values observed in the fragmentation spectra of the labeled counterparts confirmed the identity of these fragments. In the fragmentation of the [13C3]-labeled analog, the loss of formaldehyde, either from the [13C3]-labeled aliphatic side chain or from the unlabeled aliphatic side chain, resulted in a doublet of fragments at m/z 329 and m/z 330, shifted two or three Da, respectively, with respect to the analogous fragment (m/z 327) from the unlabeled compound. In the fragmentation of the [13C6]-labeled analog, the

loss of formaldehyde from either of the two [13C3]-labeled aliphatic side chains resulted in a single fragment at m/z 332, shifted five Da with respect to the analogous fragment from the unlabeled compound. Similarly, the loss of formic acid resulted in a fragment at m/z 311 for the unlabeled compound; a doublet of fragments at m/z 313 and m/z 314 for the [13C3]-labeled compound, depending on whether the carbon was expelled from the [13C3]-labeled or unlabeled aliphatic chain; and a single fragment at m/z 316 in the fragmentation of the [13C6]-labeled analog. Finally, the product ions at m/z 151 and m/z 136, which contain one aliphatic carbon atom, were represented by two doublets at m/z 151-152 and at m/z 136-137 in the spectrum of the [13C3]-labeled analog, indicating that the fragments could be derived either from the labeled or from the unlabeled end of the molecule, and were represented by single fragments at m/z 152 and m/z 137 in the spectrum of the [13C6]-labeled analog, where both ends were labeled.

References

Carroll JA, Willard D, Lebrilla CB. 1995. Energetics of cross-ring cleavages and their relevance to the linkage determination of oligosaccharides. *Analytica Chimica Acta,* **307**: 431-447.

Dauwe R, Morreel K, Goeminne G, Gielen B, Rohde A, Van Beeumen J, Ralph J, Boudet AM, Kopka J, Rochange SF. 2007. Molecular phenotyping of lignin-modified tobacco reveals associated changes in cell-wall metabolism, primary metabolism, stress metabolism and photorespiration. *The Plant Journal,* **52**: 263-285.

Debrauwer L, Paris A, Rao D, Fournier F, Tabet J-C. 1992. Mass spectrometric studies on 17β-estradiol-17-fatty acid esters: Evidence for the formation of anion-dipole intermediates. *Organic Mass Spectrometry,* **27**: 709-719.

Dima O, Morreel K, Vanholme B, Kim H, Ralph J, Boerjan W. 2015. Small glycosylated lignin oligomers are stored in Arabidopsis leaf vacuoles. *Plant Cell,* **27**: 695-710.

Eklund PC, Backman MJ, Kronberg LA, Smeds AI, Sjoholm RE. 2008a. Identification of lignans by liquid chromatography-electrospray ionization ion-trap mass spectrometry. *J Mass Spectrom,* **43**: 97-107.

Eklund PC, Backman MJ, Kronberg LA, Smeds AI, Sjöholm RE. 2008b. Identification of lignans by liquid chromatography-electrospray ionization ion-trap mass spectrometry. *J Mass Spectrom,* **43**: 97-107.

Fournier F, Perlat M-C, Tabet J-C. 1995. Control of internal proton transfers on ion-dipole complexes from [M H]− ions of diphenol esters. *Rapid Communications in Mass Spectrometry,* **9**: 13-17.

Fournier F, Remaud B, Blasco T, Tabet JC. 1993. Ion-dipole complex formation from deprotonated phenol fatty acid esters evidenced by using gas-phase labeling combined with tandem mass spectrometry. *J Am Soc Mass Spectrom,* **4**: 343-51.

Guo H, Liu AH, Ye M, Yang M, Guo DA. 2007. Characterization of phenolic compounds in the fruits of Forsythia suspensa by high-performance liquid chromatography coupled with electrospray ionization tandem mass spectrometry. *Rapid Commun Mass Spectrom,* **21**: 715-29.

Hanhineva K, Rogachev I, Aura A-M, Aharoni A, Poutanen K, Mykkänen H. 2012. Identification of novel lignans in the whole grain rye bran by non-targeted LC–MS metabolite profiling. *Metabolomics,* **8**: 399-409.

Huang S, Zhang H, Qin X, Nie C, Yu X, Deng Q. 2021. The quality and antioxidant elucidation of germinated flaxseed treated with acidic electrolyzed water. **9**: 6031-6046.

March RE, Stadey CJ. 2005. A tandem mass spectrometric study of saccharides at high mass resolution. *Rapid Commun Mass Spectrom,* **19**: 805-12.

Morreel K, Dima O, Kim H, Lu F, Niculaes C, Vanholme R, Dauwe R, Goeminne G, Inzé D, Messens E, Ralph J, Boerjan W. 2010. Mass Spectrometry-Based Sequencing of Lignin Oligomers. *Plant Physiology,* **153**: 1464-1478.

Morreel K, Saeys Y, Dima O, Lu F, Van de Peer Y, Vanholme R, Ralph J, Vanholme B, Boerjan W. 2014. Systematic structural characterization of metabolites in Arabidopsis via candidate substrate-product pair networks. *Plant Cell,* **26**: 929-45.

Mulroney B, Barrie Peel J, Traeger JC. 1999. Theoretical study of deprotonated glucopyranosyl disaccharide fragmentation. *Journal of Mass Spectrometry,* **34**: 856-871.

Nguyen TK, Jamali A, Grand E, Morreel K, Marcelo P, Gontier E, Dauwe R. 2017. Phenylpropanoid profiling reveals a class of hydroxycinnamoyl glucaric acid conjugates in Isatis tinctoria leaves. *Phytochemistry,* **144**: 127-140.

Ricci A, Fiorentino A, Piccolella S, Golino A, Pepi F, D'Abrosca B, Letizia M, Monaco P. 2008. Furofuranic glycosylated lignans: a gas-phase ion chemistry investigation by tandem mass spectrometry. *Rapid Commun Mass Spectrom,* **22**: 3382-92.

Stroobant V, Rozenberg R, el Bouabsa M, Deffense E, de Hoffmann E. 1995. Fragmentation of conjugate bases of esters derived from multifunctional alcohols including triacylglycerols. *Journal of the American Society for Mass Spectrometry,* **6**: 498-506.

Thiombiano B, Gontier E, Molinie R, Marcelo P, Mesnard F, Dauwe R. 2020. An untargeted liquid chromatography–mass spectrometry-based workflow for the structural characterization of plant polyesters. *The Plant Journal,* **102**: 1323-1339.

Vanholme R, Ralph J, Akiyama T, Lu F, Pazo JR, Kim H, Christensen JH, Van Reusel B, Storme V, De Rycke R, Rohde A, Morreel K, Boerjan W. 2010. Engineering traditional monolignols out of lignin by concomitant up-regulation of F5H1 and down-regulation of COMT in Arabidopsis. *Plant J,* **64**: 885-97.

Ye M, Yan Y, Guo DA. 2005. Characterization of phenolic compounds in the Chinese herbal drug Tu-Si-Zi by liquid chromatography coupled to electrospray ionization mass spectrometry. *Rapid Commun Mass Spectrom,* **19**: 1469-84.
