# Supplementary material for: Combining Stable Isotope Labeling and Candidate Substrate–Product Pair Networks Reveals Lignan, Oligolignol, and Chicoric Acid Biosynthesis in Flax Seedlings (Linum usitatissimum L.)
Source: Plants (Basel). 2025 Aug 1;14(15):2371. doi: 10.3390/plants14152371 (PMC12349070; doi:10.3390/plants14152371)
Supplement: Supplementary file 1 [file plants-14-02371-s001.zip › SupplementaryTableS1.pdf]

Supplementary Table S1. Mass spectral data of structurally elucidated phenylpropanoid derivatives

Nr, compound number; RT, retention time expressed in minutes; observed m/z, mean of peak m/z weighted (by intensity) across scans and averaged over different samples; formula, estimated bruto formula; Δppm, difference between observed m/z and theoretical mono-isotopic m/z corresponding to the estimated bruto formula, expressed in parts per million; SIL, stable isotope labeled analogs detected; MSn, mass spectral fragmentation obtained at nominal mass resolution (ion trap), relative intensities of fragment ions are indicted between brackets.

Nr	RT	Compound	adduct	observed m/z	formula	Δ ppm	SIL	MSn [unlabeled]	MSn [13C3]	MSn [13C6]
1	2	Caffeoyl tartaric acid	$[M-H]^-$	311.0402	C13H11 O9	-0.2	-	MS2: 149(100), 179(64), 178(4.5), 135(4.3)		
								MS3[149]: 103(100), 87(87), 131(60), 59(17), 105(3), 73(1)		
								MS3[179]: 135(100)		
2	2. 2	Protocatechoyl glucose	$[M-H]^-$	315.0713	C13H15 O9	-0.9	-	MS2: 153(100), 152(40), 165(15), 163(10), 109(9), 225(8), 151(7), 108(6)195(5), 207(4), 179(3), 181(2.6)		
								MS3[153]: 109		
								MS3[152]: 108		
3	2. 8	Hydroxybenzoic acid glucoside	$[M+Ac-H]^-$	359.0977	C15H19 O10	-0.3	[13C1]	MS2: 299(100), 137(3)		
								MS3[299]: 137(100), 93(4)		
								MS4[137]: 93		
4	3. 1	Gallic acid + glucose	$[M-H]^-$	331.0662	C13H15 O10	-0.8	-	no MS2 data		
5	3. 1	Coumaroyl tartaric acid	$[M-H]^-$	295.0453	C13H11 O8	-0.1	[13C3]	MS2 : 163(100), 113(5), 119(2)	no MS2 data	
								MS3[163]: 119(100)		
								MS3 [113]: 85(100)		
6	3. 5	Coumaroyl tartaric acid	$[M-H]^-$	295.0454	C13H11 O8	-0.1	[13C3]	MS2 : 163(100), 113(7), 119(2)	MS2: 166(100), 113(5), 121(2)	
								MS3[163]: 119	MS3[166]: 121	
								MS3[113]: 85	MS3[113]: 85	
7	3. 6	Coumaroyl glucaric acid	$[M-H]^-$	355.0662	C15H15 O10	-0.9	[13C3]	MS2: 191(100), 209(31), 337(11), 147(4), 173(2)	MS2: 191(100), 209(30), 340(10), 147(3), 173(3)	
								MS3[191]: 85(100), 147(24), 173(9), 129(7)	MS3[191]: 85(100), 147(18), 173(8), 129(8)	

								MS3[209]: 191(100), 85(9), 147(3)	MS3[209]: 191(100), 85(8), 147(4), 173(2)	
								MS4[85]: 57	MS4[85]: 57	
8	6.2	Coumaric acid glucoside glucose ester	[M+Ac-H]$^-$	547.1659	C23H31O15	-0.8	[13C3]	MS2: 487(100), 325(72), 163(2)	MS2: 490(100), 328(82), 370(4), 400(3), 430(2), 182(2), 166(2)	
									MS3[490]: 328(100), 166(57)	
9	6.2	Caffeic acid glucoside	[M-H]$^-$	341.0871	C15H17O9	-0.5	[13C3]	MS2: 179(100), 135(4)	MS2: 182(100), 137(4)	
								MS3[179]: 135	MS3[182]: 137	
10	6.9	Coumaric acid glucoside	[M+Ac-H]$^-$	385.1131	C17H21O10	-1	[13C3]	MS2: 325(100), 163(49)	MS2: 328(100), 166(51)	
								MS3[325]: 163(100), 119(3)	MS3[328]: 166(100), 121(4)	
11	8.5	Ferulic acid glucoside	[M+Ac-H]$^-$	415.1234	C18H23O11	-1.5	[13C3]	MS2: 355	MS2: 358	
								MS3[355]: 193(100), 178(3)	MS3[358]: 196(100), 151(4)	
									MS4[196]: 151(100), 181(64), 136(39)	
12	8.5	Dihydro-p-coumaroyl glucose	[M-H]$^-$	327.1077	C15H19O8	-0.9	[13C3]	MS2: 165(100), 121(2)	MS2: 168(100), 123(2)	
								MS3[165]: 121(100), 93(28), 59(2)	MS3[168]: 123(100), 93(7), 61(7)	
13	8.8	Coumaroyl glucose	[M-H]$^-$	325.0924	C15H17O8	0.1	[13C3]	no MS2 data	MS2: 166(100), 148(99), 190(57), 268(23), 121(11), 238(11), 208(9), 267(7), 165(4), 220(3)	
14	8.9	Coniferin	[M+Ac-H]$^-$	401.1447	C18H25O10	-0.1	[13C3]	MS2: 179(100), 341(30), 164(3), 161(3)	no MS2 data	
								MS3[179]: 161(100), 164(59), 146(25)		
								MS3[130]: 179(100), 164(2), 161(2)		
								MS4[161]: 146		
15	9	Lariciresinol diglucoside	[M+Ac-H]$^-$	743.2749	C34H47O18	-1.8	[13C3], [13C6]	no MS2 data	no MS2 data	no MS2 data
16	9.4	Chicoric acid	[M-H]$^-$	473.0718	C22H17O12	-0.4	[13C3], [13C6]	MS2: 311(100), 293(98), 149(10), 179(9)	MS2:314(100), 311(99), 296(96), 293(93), 149(18), 313(13), 295(12), 179(10)	no MS2 data
								MS3[311]: 149(100), 179(60), 135(2)	MS3[314]: 149(100), 182(60), 181(4), 137(3)	

								MS3[293]: 219(100), 275(26), 113(7), 231(7), 139(6)	MS3[311]: 149(100), 179(53), 135(3)	
								MS4[149]: 87(100), 103(98), 131(68), 59(24)		
17	9.6	Coumaric acid	[M-H]-	163.0398	C9H7O3	1.8	[13C3]	MS2: 119(100)	MS2: 121(100)	
18	10.3	Coumaroyl caffeoyl tartaric acid	[M-H]-	457.077	C22H17O11	-0.1	[13C3], [13C6]	MS2: 295(100), 293(68), 277(52), 163(7), 179(3)	no MS2 data	no MS2 data
								MS3[295]: 163(100), 113(4), 149(2)		
								MS3[293]: 219(100), 275(22), 231(8), 139(7), 113(6), 221(2), 127(2)		
								MS4[163]: 119		
19	10.5	Lariciresinol diglucoside	[M+Ac-H]-	743.2744	C34H47O18	-2.5	[13C3], [13C6]	no MS2 data	no MS2 data	no MS2 data
20	10.6	G(8-O-4)G glucoside	[M+Ac-H]-	597.2165	C28H37O14	-4.0	[13C3], [13C6]	MS2: 375(100), 537(42)	MS2: 540(100), 378(88)	MS2: 381(100), 543(75)
								MS3[375]: 327(100), 195(15), 179(4), 165(4), 357(1)		
								MS4[327]: 312(100), 280(19), 164(9)		
21	11.4	Pinoresinol diglucoside	[M+Ac-H]-	741.2587	C34H45O18	-2.6	[13C3], [13C6]	MS2: 681(100), 501(68), 609(68), 643(68), 568(53), 684(50), 724(44), 361(38), 706(35), 579(29), 694(29), 458(20), 463(12), 697(12)	no MS2 data	no MS2 data
								MS3[681]: 519(100), 357(27), 637(3), 664(2)		
								MS4[519]: 357(100)		
								MS4[357]: 151(100), 342(30), 311(10), 136(9), 175(5)		
22	11.4	Dicoumaroyl tartaric acid	[M-H]-	441.0816	C22H17O10	-1.2	[13C3], [13C6]	MS2: 277(100), 295(4), 203(3)	no MS2 data	MS2: 280(100), 298(39), 206(4)
								MS3[277]: 203(100),233(29), 259(20), 205(4)		MS3[280]: 206(100), 236(36), 262(25), 218(4), 121(1), 148(1), 113(1), 131(0.5)
										MS3[298]: 166(100), 113(5), 149(2), 121(1), 103(0.5), 131(0.5)

										MS4[206]: 177(100), 178(53), 149(19), 147(2)
23	11.8	Hydroxyphenyl acetic acid	[M-H]$^-$	151.0399	C8H7O3	2.3		MS2: 107(100), 77(6), 105(4), 93(4)		
							-	MS3[107]: 77(100)		
24	12.6	Dicoumaroyl tartaric acid	[M-H]$^-$	441.0816	C22H17O10	-1.2	[13C3], [13C6]	MS2: 277(100), 295(36), 203(3)	no MS2 data	MS2: 280(100), 298(38), 206(4)
								MS3[277]: 203(100),233(33), 259(22), 215(3), 205(3), 145(2), 131(1), 113(1), 119(0.5)		MS3[280]: 206(100), 236(27), 262(25), 218(4), 121(1), 148(0.5), 131(0.5), 113(0.5)
								MS3[295]: 163(100), 113(5), 149(2), 119(2), 131(1), 103(1)		MS3[298]: 166(100), 113(7), 149(2), 121(2), 103(0.5), 131(0.5)
								MS4[203]: 175(100), 147(8)		MS4[206]: 177(100), 178(39), 149(11), 147(1)
25	12.6	Dehydrodiconiferyl alcohol hexoside	[M+Ac-H]$^-$	579.2074	C28H35O13	-0.7	[13C3], [13C6]	MS2: 339(100), 327(60), 357(22), 417(9), 519(5), 387(3), 356(2)	no MS2 data	MS2: 345(100), 332(57), 363(17), 362(5), 525(4), 330(2)
								MS3[339]: 324(100), 307(6), 309(6), 308(3), 179 (1)		MS3[345]: 330(100), 313(7), 315(6), 314(4), 182(1)
								MS3[327]: 309(100), 312(27), 297(18), 294(6), 283(4)		MS3[332]: 314(100), 317(25), 301(17), 299(7), 286(5), 283(3)
								MS4[324]: 309(100), 295(2)		MS4[330]: 315(100), 302(2), 299(2), 312(1)
26	12.9	Lariciresinol glucoside	[M-H]$^-$	521.2015	C26H33O11	-1.6	[13C3], [13C6]	MS2: 329(100), 359(54)	no MS2 data	MS2: 334(100), 365(59), 483(2)
								MS3[329]: 178(100), 299(56), 193(23), 192(19), 160(18), 175(17), 314(17), 284(13), 161(13), 159(8), 123(7), 177(5), 174(4), 311(3), 269(3), 121(2)		MS3[334]: 182(100), 303(56), 197(22), 196(17), 179(14), 165(13), 319(13), 163(9)
								MS3[359]: 329		MS3[365]: 334(100), 348(3)
								MS4[178]: 160(100), 161(41), 159(18), 122(9), 147(6)		MS4[182]: 164(100), 163(28), 165(8), 123(6)
27	13.9	Pinoresinol monoglucoside	[M-H]$^-$	519.1865	C26H31O11	-0.2	[13C3], [13C6]	MS2: 357(100)	MS2: 360(100),359(2), 358(1)	MS2: 363
								MS3 [357]: 151(100), 136(37), 311(14), 342(12), 175(4), 327(3), 137(3)	MS3[360]: 151(100), 152(65), 137(38), 136(36), 345(19), 313(16), 314(16), 178(5)	MS3[363]: 152(100), 137(32), 316(19), 348(13), 332(4), 179(3), 138(2), 211(2), 164(1), 180(1)
								MS4[151]: 136(100)	MS4[151]: 136(100), 123(2)	MS4[152]: 137(100)
