# Supplementary figures and images for: Combining Stable Isotope Labeling and Candidate Substrate–Product Pair Networks Reveals Lignan, Oligolignol, and Chicoric Acid Biosynthesis in Flax Seedlings (Linum usitatissimum L.)
Source: Plants (Basel). 2025 Aug 1;14(15):2371. doi: 10.3390/plants14152371 (PMC12349070; doi:10.3390/plants14152371)

C

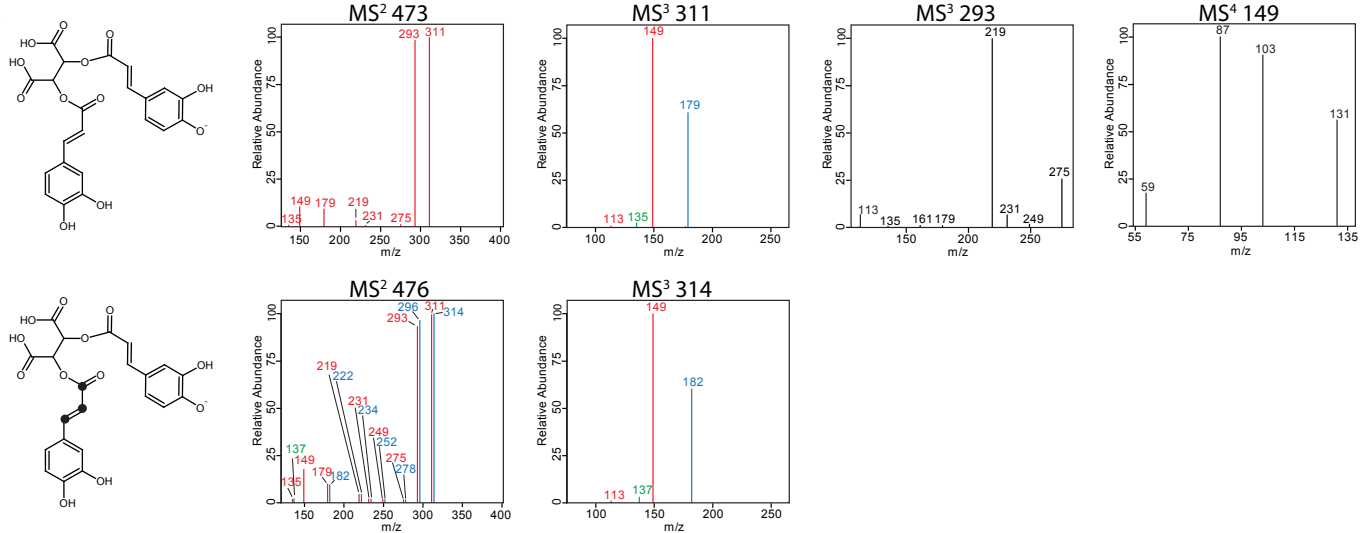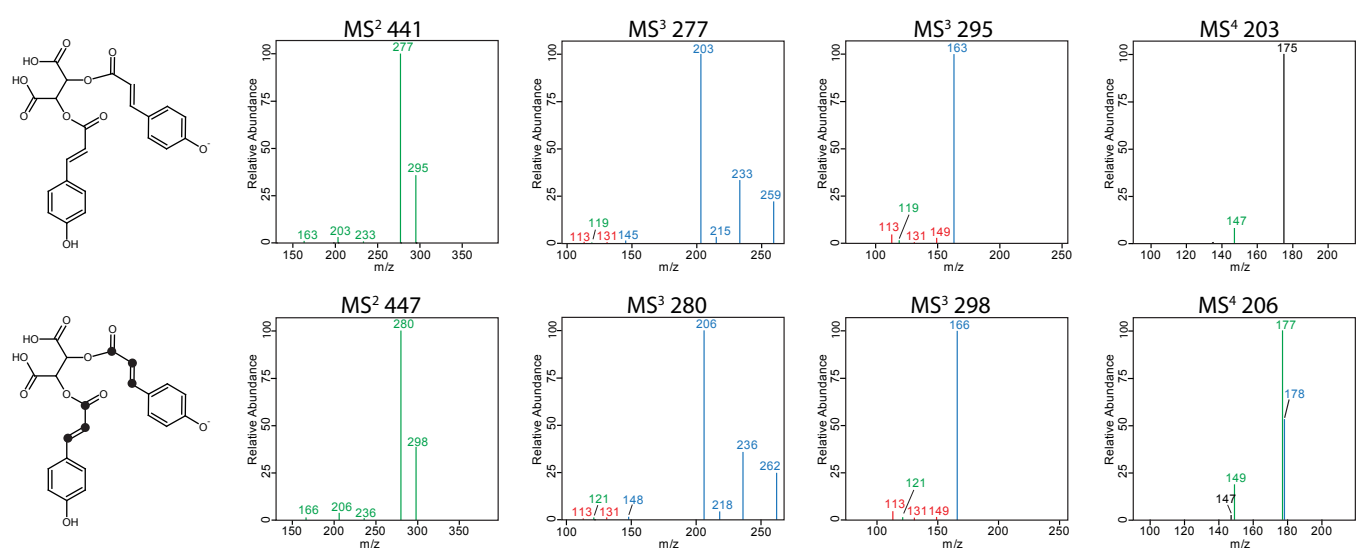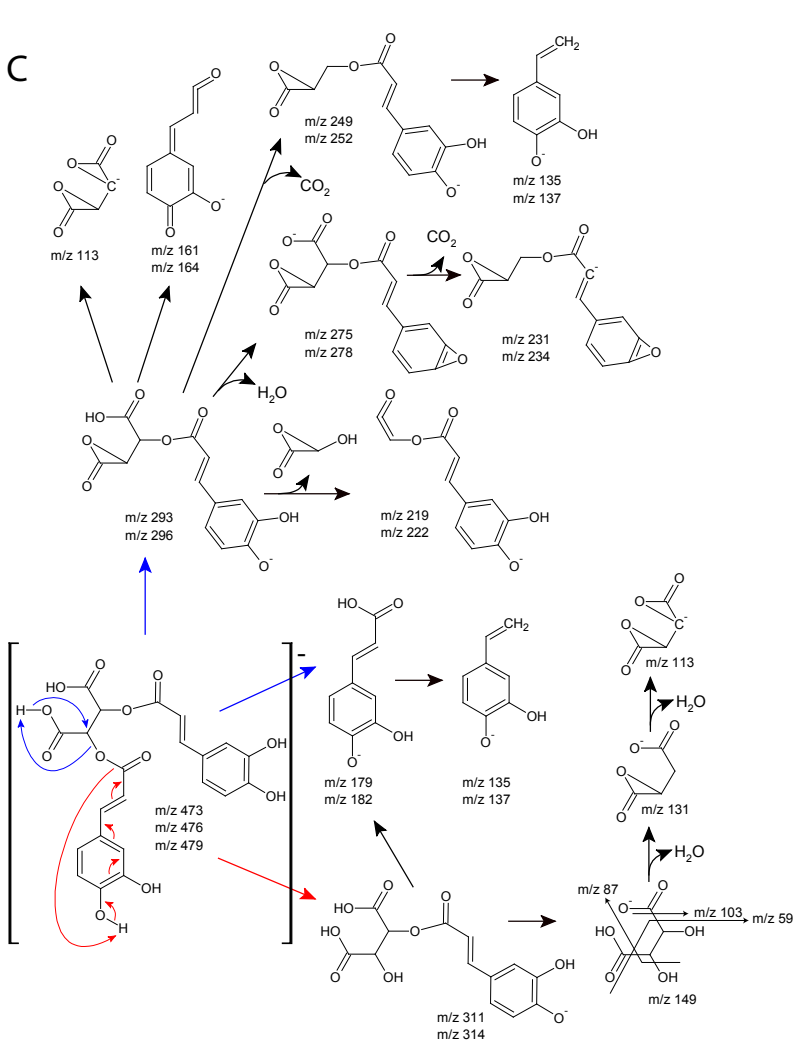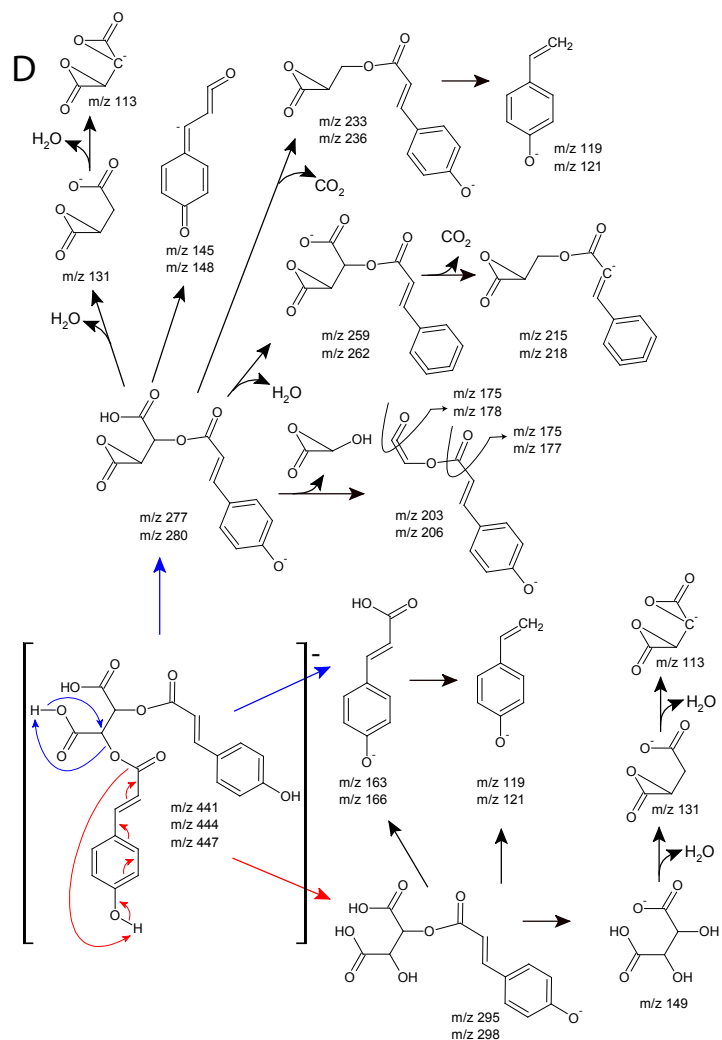

Supplement: Supplementary file 1 [file plants-14-02371-s001.zip › SupplementaryFigureS1.pdf]

Clusters' members (z-normalized)

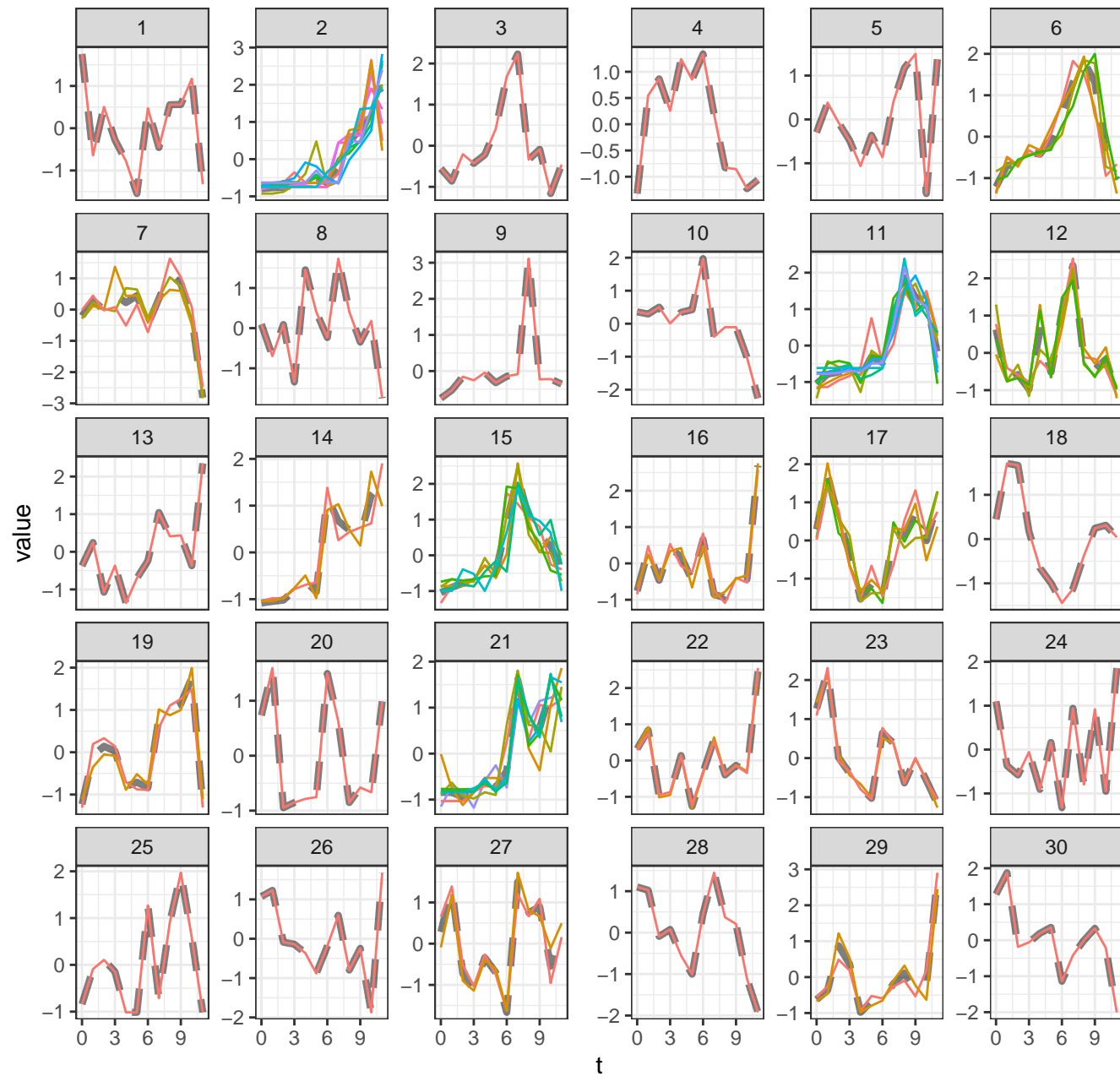

Supplement: Supplementary file 1 [file plants-14-02371-s001.zip › SupplementaryFigureS2.pdf]
